# The management of good manufacturing practice (GMP) inspections: a scoping review of the evidence

**DOI:** 10.3389/fmed.2025.1687864

**Published:** 2025-11-11

**Authors:** Aisha Al Azawei, Kate Loughrey, Kim Surim, Mary Elizabeth Connolly, Bernard D. Naughton

**Affiliations:** 1 School of Pharmacy and Pharmaceutical Sciences, Trinity College Dublin, Dublin, Ireland; 2 Centre for Pharmaceutical Medicine Research, Kings College, London, United Kingdom

**Keywords:** pharmaceutical, good manufacturing practice, good inspection practices, inspection, management, pharmaceutical industry, GMP, pharmaceutical quality

## Abstract

**Introduction:**

Regulatory agencies impose stringent legislation and guidelines in the pharmaceutical industry to ensure the safety and quality of medicinal products. While most regulatory research focuses on market authorization, there are fewer in-depth discussions covering production management. This scoping review explores how good manufacturing practices (GMP) inspections are conducted and identifies best practices for managing them across several phases.

**Methods:**

This review adhered to the Joanna Briggs Institute guidance for scoping reviews. The SPIDER framework was utilized for qualitative evidence synthesis to develop the inclusion criteria of this study. The chosen databases were PubMed and Embase (English only, 2015–2025), and grey literature was identified via Google Advanced.

**Results:**

Of 377 sources screened, 74 sources (19.63%) met the inclusion criteria: 14 academic papers and 60 grey literature sources. Thematic analysis identified key strategies for managing GMP inspections across three phases: pre-inspection, execution, and post-inspection. These findings could improve industry compliance, streamline inspection readiness, and reduce uncertainties. These findings are particularly beneficial in low- and middle-income countries where regulatory frameworks are often less evolved. Improving GMP inspection management can contribute to upholding patient safety.

**Conclusion:**

GMP inspection management is an unaddressed topic, and further research could deliver value to regulators and manufacturers. The data in this paper offers recommendations, including a checklist to support GMP inspections, which may be useful to international manufacturers either in its current state or as a template that can be adapted to specific company contexts. This study highlights the need for advanced inspection methodologies, greater transparency, and stronger collaboration between regulators and manufacturers to safeguard public health through what we describe as ‘Good Inspection Practices’(GIP).

## 1 Introduction

Good manufacturing practice (GMP) inspections are of fundamental importance to the pharmaceutical industry. They ensure high-quality standards for medicinal products before they reach the market. As part of GMP, manufacturers must identify and address deficiencies in their quality management systems to ensure patient safety and avoid penalties (1). Regulatory authorities, including the European Medicines Agency (EMA), the Food and Drug Administration (FDA), and the World Health Organization (WHO), provide guidelines to support GMP compliance and safeguard public health (2–4). However, implementing these guidelines from a manufacturer’s perspective and enforcing them from the regulatory side can lead to discrepancies between theoretical expectations and practical execution.

The inspection process involves a review of a company’s premises, procedures, processes, people, and products; widely known as the ‘5 P’s’. Premises refer to the physical areas where manufacturing happens, procedures are the official, verified and documented ways of working, processes are the repeatable activities which ensure consistency, compliance, and quality throughout operations and are governed by standard operating procedures (SOPs). At the core of those four components lie the people who drive the execution, compliance, oversight, and continuous improvement of GMP practices. Finally, product review includes the assessment of product quality according to company evidence. Poor inspection results and product quality problems can lead manufacturers to halt production and expend resources on investigating the root cause. This can lead to drug shortages and treatment delays, resulting in losses for the company and the patient. However, quality breaches are often undetected until after product release, highlighting the need to develop strategies to tackle this problem (5, 6).

A study that analyzed 99 GMP inspection reports across 19 countries over 10 years identified 1,458 deficiencies; 37% were major and 9% were critical (1). Another study revealed a 40% decrease in inspections, from 1,748 in 2018 to 1,106 inspections in 2020 (7), demonstrating the value of robust compliance and ongoing regulatory oversight. Achieving compliance is not solely the regulator’s responsibility; the company’s personnel play a key role in ensuring high quality (8).

Inspection management or readiness is a rarely discussed but important part of a company’s quality system. A well-managed GMP inspection process involves: preparation for inspection, engagement during inspection, and post-inspection follow-up. Pre-inspection involves staff training, facility organization, documentation review, equipment handling, and risk assessment (6, 9). During inspections, inspectors evaluate the facility, compliance with standard operating procedures (SOPs), and adherence to data integrity protocols (10–12). Post-inspection, companies must respond to findings, implement corrective actions, and monitor ongoing compliance to prevent future violations (13, 14). However, there is no standardized set of frameworks for managing these inspections.

This study aimed to examine the scope and volume of evidence available concerning GMP inspections within the pharmaceutical industry, focusing on reporting current practices, challenges encountered, and opportunities for improvement. The objectives of this scoping review are to:

- Systematically analyze critical GMP and inspection mechanisms.
- Map practices used across different establishments in executing GMP.
- Understand how best to improve GMP inspection practices and management.
- Describe how a pharmaceutical company prepares for an inspection.
- Understand arrangements during and after an inspection.
- Assess how limitations in GMP inspection management by a company may influence outcomes.
- Propose a framework to guide GMP inspections (15).

## 2 Methods

### 2.1 Study design

A scoping review methodology was chosen due to the broad nature of the research question. This approach was systematic in terms of identifying, extracting, and analyzing literature from multiple databases (15). A qualitative research approach guided the study selection process.

### 2.2 Identify research question

We used the sample, phenomenon of interest, design, evaluation, and research type (SPIDER) framework to guide the development of the research question (Table 1) (16).

**Table 1:** Framework for determining the eligibility of the research question.

Sample	Companies and inspectors involved in good manufacturing practice inspections.
Phenomenon of interest	The management practices, outcomes, gaps, challenges, and opportunities associated with GMP inspections.
Design	Analysis of academic literature, grey literature, and regulatory literature related to GMP inspections.
Evaluation	Opportunities and challenges in GMP inspection management, variations in inspection practices, and prospective ways to enhance these management strategies
Research type	Qualitative research

### 2.3 Search strategy

This study conducted a comprehensive literature search across several databases. Search terms and synonyms were developed, Boolean operators were used, and filters were applied under the guidance of the institute’s subject librarian. Relevant articles were identified from academic and grey literature. Academic sources were obtained from EMBASE and PubMed, while Google Advanced was used to access grey literature.

Tables were developed to summarize figures, search dates, relevant databases, and search strings for academic literature. The number of retrieved items, search dates, organization names, and grey literature webpage links were recorded (17). Our primary research methodology, study selection, and quality assessment were guided by the PRISMA guidelines for the scoping review checklist (18, 19). The detailed academic literature search strategy, including databases used, search dates, strings, Boolean operators, and filters applied, is listed in Supplementary File 1: Table 1, while the grey literature search strategy is listed in Supplementary File 2: Table 4 (17).

### 2.4 Eligibility criteria

The eligibility criteria were developed to address the main focus of our study, which was key aspects of good manufacturing practice (GMP) inspection management.

#### 2.4.1 Inclusion criteria

This study’s inclusion criteria are:

- Studies that address GMP inspection management due to our choice of a scoping review, including the technical and management aspects of inspections.
- Academic literature databases, including Embase and PubMed.
- English language publications.
- Articles published from 1 January 2015 to 1 January 2025 are included due to their relevance to current GMP practices.
- GMP inspections within the pharmaceutical industry.
- Peer-reviewed articles and grey literature.
- Grey literature included documents from professional private companies and government organizations such as the European Medicines Agency, the World Health Organization (WHO), and the Health Products Regulatory Authority (HPRA). These sources included relevant reports, guidelines, and policy documents, such as technical reports, institute standards, research reports, conference papers, and opinion pieces, produced by academic, government, and professional organizations.
- Any geographic location.

#### 2.4.2 Exclusion criteria

The excluded studies were:

- Studies that discuss specific products of GMP, such as radiopharmaceuticals and biologic medicines, but not inspection management.
- Articles discussing GMP practices not related to inspections.
- Publications before 2015.
- Non-English language publications.
- Hospitals or other non-pharmaceutical industry-related settings.

### 2.5 Study selection

Once the search strategy was established (Supplementary File 1: Table 1), the studies were screened, duplicates were removed, and data were extracted from academic literature using Covidence (20) (Supplementary File 1: Table 2). Covidence supported the management and review of studies in this project. Grey literature screening was carried out separately. Duplicates were identified and removed manually, and studies were excluded based on the inclusion criteria. To ensure credibility and methodological transparency, a structured assessment approach was used. For grey literature, sources were screened based on the reputation of the issuing organization (regulatory bodies, government agencies, or recognized professional organizations), the publication’s date, and its relevance to current GMP practices. Documents lacking methodological clarity and organizational affiliation were excluded to enhance the reliability and credibility of the grey literature. Further details of this process are listed in Supplementary File 2: Table 4. For academic databases, articles were imported from EndNote into Covidence for screening (21). Study selection took place in three stages. Four reviewers independently screened articles, with each article undergoing double screening. The first stage involved screening the title and abstract and removing duplicates. Conflicts were resolved through discussion, and a third reviewer was consulted to reach the final decision.

The second stage involved a full-text review to screen and decide whether to include or exclude studies based on our eligibility criteria. Discrepancies were resolved in the same way as in the first stage. The third stage involved data extraction from the final set of included articles.

For grey literature, the total number of results was gathered according to the same inclusion criteria and imported into a shared document. These results were then divided among the four reviewers for initial screening, followed by a secondary review. If discrepancies arose, a third reviewer (BN) was consulted to reach a consensus.

### 2.6 Data processing

#### 2.6.1 Data extraction

Qualitative data were captured using a structured data extraction form (Supplementary File 1: Table 2). Covidence and Microsoft Excel were used to support this process and to track academic literature throughout the screening and extraction stages (20, 22). The extraction form was guided by the SPIDER framework (16).

For academic studies, the following data were extracted:

- Study title
- Author(s)
- Year of publication
- Country
- Study design
- Data collection method
- Data analysis method
- Study aim
- Reported outcomes
- Reported limitations

The final extraction table is presented in Supplementary File 1: Table 2.

For grey literature, data extraction was conducted independently by a single reviewer; it included:

- Year of publication
- Country
- Aims or objectives
- Outcomes
- Type of source (e.g., policy report)

Further details are shown in Supplementary File 2 Table 4.

#### 2.6.2 Data synthesis

Thematic analysis was used to synthesize academic and grey literature findings. The data were coded using Delve, a qualitative analysis software. Main themes were generated inductively and grouped according to the phases of GMP inspections (pre-inspection, execution, and post-inspection). These were further refined into subthemes for deeper analysis of practices, challenges, and opportunities related to GMP inspection management (23).

### 2.7 Quality appraisal of included studies

The use of a risk-of-bias assessment tool is optional in scoping reviews. However, the Mixed Methods Appraisal Tool (MMAT) version 2018 (24) was used to assess the reliability, validity, and quality of the academic studies included. The assessment involved answering two initial screening questions, followed by five core criteria with ‘yes’, ‘no’, or ‘cannot tell’. A scoring system was used to evaluate the quality of each study (25). One point was awarded when a criterion was answered with ‘yes’, and the total score of five determined the study’s quality. Two points or less suggested low quality, three points suggested moderate quality, four points suggested good quality, and five points suggested excellent quality of the selected study. The grey literature sources did not undergo a quality appraisal. Nonetheless, assessing the quality of academic evidence was important in mapping the strengths and limitations of included studies (Supplementary File 1: Table 3).

## 3 Results

### 3.1 Screening results

**Figure 1:**
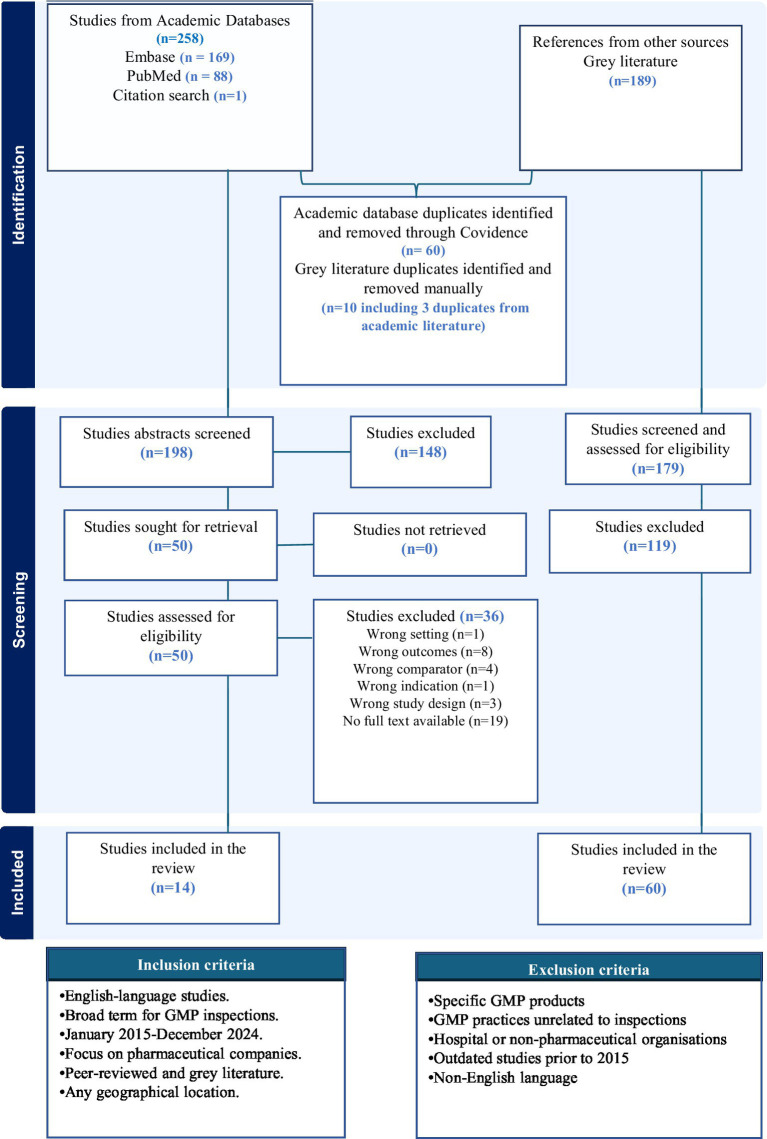
Literature search, PRISMA diagram, inclusion, and exclusion criteria.

A total of 447 papers were initially screened from academic and grey literature sources, with 70 duplicates removed across all sources. Fifty academic articles were assessed for full-text screening, of which 14 were included. For grey literature, 179 results were assessed during full-text screening, and 60 were included (Figure 1). The results for the grey literature and academic literature data extraction are presented in Supplementary File 2: Table 4 and Supplementary File 1: Table 2, respectively.

The inter-rater agreements for the title and abstract screening among reviewers ranged from slight (20%) to substantial agreement (72.7%), with Cohen’s kappa values of 0.622, 0.543, 0.727, 0.314, 0.200, and 0.227. For the full-text screening, the inter-rater agreements ranged from slight (8.6%) to perfect agreement (100%), with corresponding Cohen’s kappa values of 0.580, 0.846, 0.5, 1.0, 0.0869, and 0.545.

### 3.2 Characteristics of included studies

Characteristics of the included academic articles are outlined in Supplementary File 1: Table 2. Included studies were published between 2015 and 2025, with a majority (*n* = 8, 57%) published after 2020. Among the 14 included academic articles, eight studies focused on India (26–33), and the rest were based on Thailand (34), China (8), Bulgaria (1), the USA (5), Switzerland (35), and Pakistan (36).

This suggests a notable concentration of research from India, which influences the generalizability of the findings. Grey literature sources were more geographically diverse and primarily drawn from the UK, EU, and US regulatory guidance and reports.

### 3.3 Quality assessment of included studies

A total of 13 out of 14 academic studies passed the initial screening questions and were subsequently assessed using the five core MMAT criteria. The study that did not pass the initial screening lacked a clearly defined research question and did not indicate that the collected data were intended to address a specific objective. Among the studies that passed the initial screening, seven were rated as low quality, four were rated as moderate quality, one was rated as good quality, and one was rated as excellent quality. The overall average indicates that the majority of the studies in this research area are of low quality (Supplementary File 1: Graph 1 and Supplementary File 1: Table 3).

### 3.4 Study findings

Inspection process: Based on data from this study, manufacturers are typically given 4–6 weeks’ notice before an inspection. However, unannounced inspections may also be conducted when necessary. EMA inspections can be carried out over 3–5 days, while FDA inspections can last for 2 weeks or longer. There can be extensive pre-inspection requests, within inspection requests and document requirements. Inspections, in simple terms, consist of an opening meeting, document review, walk-through of the facilities, sessions with key personnel, and a closing meeting (10).

**Figure 2:**
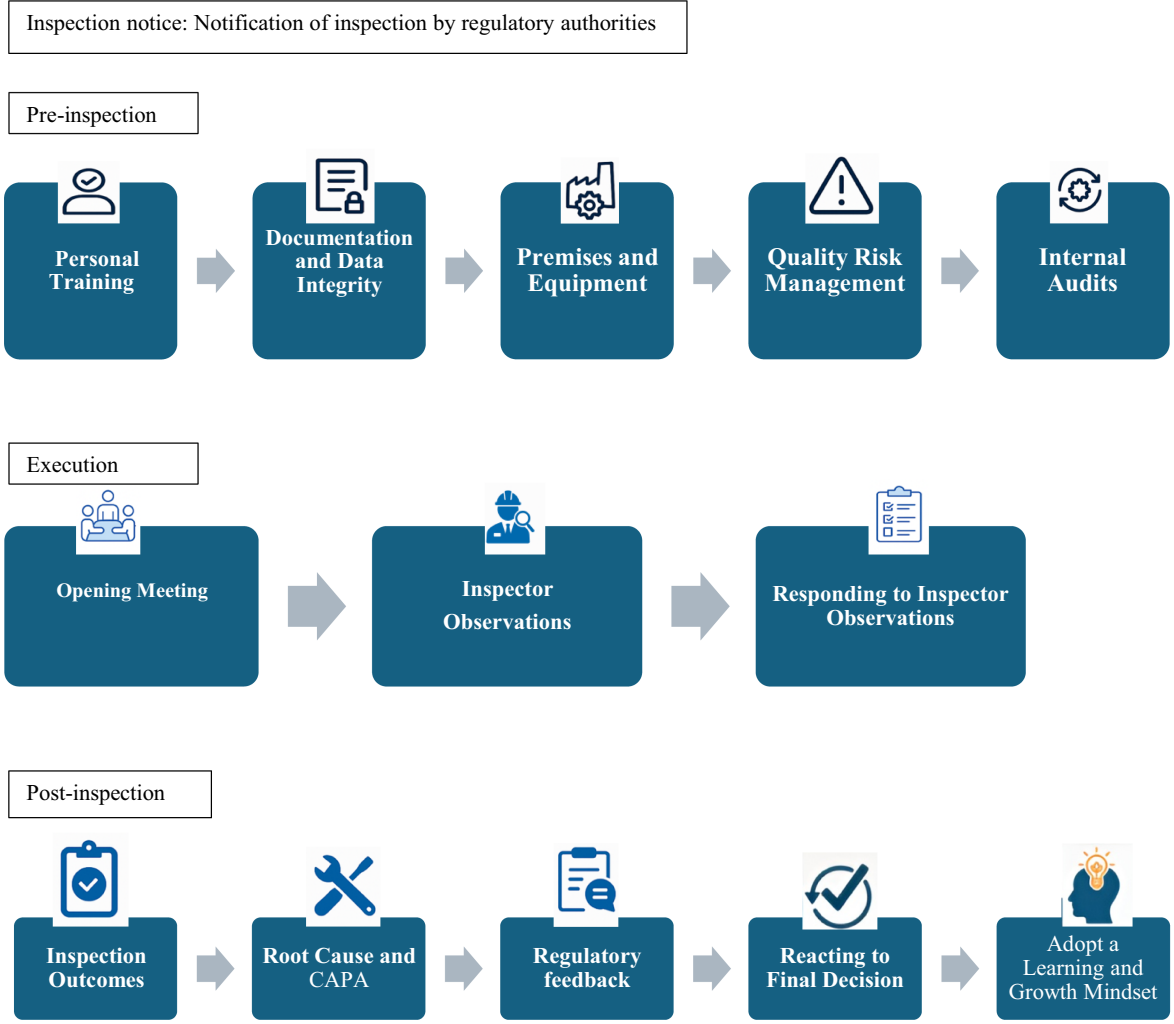
Results of thematic analysis.

The following three themes emerged from analyzing academic and grey literature, as outlined in Figure 2:

1. The pre-inspection phase (preparation),
2. During the inspection phase (inspection execution),
3. The post-inspection phase (follow-up)

#### 3.4.1 Pre-inspection phase (preparation)

This phase discusses actions that companies and regulatory authorities should consider when preparing for an inspection. Companies could begin by conducting an overall evaluation of inspection readiness (29). They take into consideration factors that affect GMP compliance, such as company ownership type, financial resources, bank loans, operating time, production experience, the number of batch varieties produced annually, and the company’s fixed assets; this is particularly important for smaller companies (8). After this initial evaluation, companies address key components, such as personnel training, documentation, premises and equipment, quality risk management, and internal audits (11–14, 37).

##### 3.4.1.1 Regular personnel training and education

A need for comprehensive training of personnel, which covers all aspects of production procedures (1), including change control (27), new SOPs (29), computer system validation (31), preparatory knowledge of good documentation practices (GDocPs) prior to inspector queries (33), and training on ALCOA+ principles, is essential for inspection readiness (33, 35). Staff should be educated on regulatory expectations regarding quality assurance, GMP compliance concepts, and the roles of relevant stakeholders (36). Additionally, staff should also stay informed about updates related to specific procedures, such as ICH guidelines (35). Another important key activity is fostering a ‘quality mindset’ among staff that prioritizes compliance over profit (36). Retaining quality authorized personnel with experience in the company and ensuring staff are trained before inspection has been shown to improve compliance outcomes (8).

##### 3.4.1.2 Quality management system: organize documentation and ensure data integrity

In pharmaceutical manufacturing, a QMS is required under GMP regulations, including EudraLex Volume 4, FDA 21 CFR Part 210/211, and ICH Q10.1. For businesses to guarantee high and consistent product quality, a Quality Management System (QMS) must take into account the standards outlined in the relevant GMP legislation. Companies have increasingly adopted electronic QMS (eQMS), which must undergo Computer System Validation (CSV) before its implementation. The benefits of a CSV are shown to increase compliance with GMP and enhance product quality and safety (31). It is said that a strong eQMS will define and control every aspect of the “5 P’s.” (6) It provides a consistent approach to document control, including standard operating procedures (SOPs) and training proof documents, product specifications, control manufacturing processes, and managing the maintenance of equipment and facilities (12, 26, 32). Documentation records must be maintained according to written procedures. Quality management personnel must have access to the most up-to-date version. Records may be kept in paper, electronic, and photographic formats; if handwritten, they must be clear, legible, indelible, and retained for a fixed period of time (26, 33). Employees have ethical considerations and must ensure data integrity, including authentic and trustworthy records, to avoid misleading inspectors (29, 33). Obsolete documents should be removed to reduce inspection errors (26). The EU Commission and the WHO emphasize the importance of good documentation practices (GDocPs) as part of quality assurance, encouraging a systematic approach to ensure compliance (33). There are two main types of documents: instructions and records/reports (33). These can be arranged in a hierarchical system, starting with the broader documents such as the quality manual, followed by company policies and SOPs, and ending with the more specific documents that include batch records, test methods, specifications, and validation protocols (38). Those records should be completed at the time of each operation to ensure modification recording and traceability of all activities (33). Other documents, such as market complaint forms, change control logbooks, and archived files, should be kept separately, updated as needed, and made available for inspectors (26). Companies must also prepare digital documentation for remote inspections (1). These systems should be password-protected, accessible only to authorized personnel, and capable of showing the history of changes and deletions. All records are to be retained for a specific period after the expiry date of the finished product, depending on the region under inspection (33). Mock inspections and data integrity checks have been identified as methods to address gaps in documentation practices (33, 35).

##### 3.4.1.3 Optimize premises and equipment

Premises should follow a logical layout, ideally using a design that includes sufficient working space, proper air circulation outlets, well-installed electrical wiring, and service lines (30). Facilities should meet safety and contamination prevention standards (26). Specific storage areas should be allocated for highly toxic or hazardous products (38), and access should be restricted to authorized staff (30). Rooms for documentation entries should also be provided (33). Equipment should be visually inspected (32), thoroughly washed, and checked for contamination before production starts (30). It should be maintained, serviced, modified, and tested before use. Faulty equipment should be removed from service, and storage should proceed without delays, following written protocols to ensure it is in proper condition for reuse. Clear product labelling and proper equipment organization should be ensured before inspection. Cross-contamination during packaging, labelling, and throughout the process operations should be prevented (30) by following EMA guidelines on permitted daily exposure (PDE) for contamination control (35). All of these activities should be documented and organized for the inspectors.

##### 3.4.1.4 Ensure a clear, strong quality risk management system is in place

Implementing a quality risk management (QRM) strategy leads to fewer GMP violations. QRM involves a proactive and retrospective systematic approach to assessing, controlling, and reviewing risks to product quality based on scientific knowledge and process experience (8, 39).

Companies should perform a quality risk assessment to support deviation investigations and the implementation of corrective and preventive actions (CAPA) before the inspection, which includes identifying major, minor, and critical deviations and implementing a system to prevent recurrence (29). Quality risk assessment should also be implemented and documented during the change control procedures, including classification of change by type and priority and evaluating the impact on product, process, and quality before implementation (27). QRM systems can be validated before inspection by going through a PIC/S audit checklist that covers: staff qualifications, inspection resources, training programs, and compliance policies (28).

##### 3.4.1.5 Practice and preparation: internal audits and self-inspections

Companies must identify recurring issues and deficiencies, using them as guidance to establish corrective actions and conduct internal inspections before regulatory inspections. They should prioritize risk-based self-inspections to prevent violations (1).

It is recommended to prepare for quality assessments before inspections take place by using open-ended questions for employees about their experiences and perspectives, which can be part of the internal auditing procedure (36). Internal audits could be performed at least twice a year to ensure regulatory compliance before external audits are conducted by regulatory authorities (9, 26).

Companies should conduct their internal annual quality review survey to inspect for any indication of faulty drug batches, thereby increasing their production diligence and addressing gaps in practice (8). The FDA’s six-system inspection model can be used to support inspections. This model aims to enable inspections to be more structured, comprehensive, and targeted. It involves evaluating and reviewing the QMS, production system, facility and equipment system, laboratory control system, materials system, and packaging and labelling system (32). High failure rate products, such as sterile products or biologics, require extra attention from the manufacturer (1). To aid self-inspections, companies can seek expert guidance and compare international standards to enhance compliance and expedite regulatory approval (8, 30).

From the regulatory authorities’ perspective, inspectors review a company’s compliance history and its ability to implement and sustain GMP (8). The selection of an expert panel is crucial, and qualifications and experience should be considered. This leads to adopting a methodological approach when selecting inspectors based on the insight of knowledgeable professionals from diverse backgrounds (5).

Manufacturers are typically required to submit pre-inspection documentation for review, such as the current site master file, GMP inspection reports, and process reports (10, 34). Using this information, the inspection plan can be developed 1–2 weeks before the scheduled visit (10).

#### 3.4.2 During-inspection phase (execution)

The first hours of the inspection will set the tone for the entire visit (14). On-site GMP and GDP inspections have restarted after being postponed or carried out remotely during the pandemic. From the time the inspectors arrive, it should be evident that the facility operates with professionalism (2).

##### 3.4.2.1 Opening meeting

A presentation of site updates since the regulators’ last visit, evidence of completed follow-up actions, and successful inspections from other regulatory authorities could be shared. Inspection themes may be announced, along with comments on pre-inspection material. Relevant staff should be available for interviews. After the opening meeting, inspection of additional documentation, sites, and personnel will begin. Employee responses should be concise and factual. Every comment mentioned during the inspection can be noted in the final report. If asked to explain a certain process, a structured approach that includes examples should be provided. A weak response will be informal, lack documentation, and suggest non-compliance (2, 14).

A daily summary can be requested from the inspectors so that the site can prepare any further documentation to correct misunderstandings or clarify concerns identified by the inspector. During the closing meeting, the inspector will provide initial impression feedback, including all observations (3).

*Initial Engagement*: A designated ‘war room’ should be prepared in advance and stocked with organizational charts, quality system manuals, SOPs, and staff training documentation (13). Open communication, brainstorming, and informal networking during the inspection are encouraged to maintain a cooperative and professional relationship with inspectors. Companies should foster unbiased dialogue and transparency throughout the inspection process (14, 28).

*Assess Expertise and Bias:* It is also recommended that companies verify the inspector’s qualifications and training validation, ensuring they align with the Pharmaceutical Inspection Co-operation Scheme (PIC/S) vision of no political involvement and no discrimination during inspections to uphold regulatory integrity during inspection (28). With regard to inspectors in particular regions, it may be important to ensure that they do not have financial or personal ties to the facility being inspected (8) and that they receive ongoing training in line with evolving technologies to assess suitability. They must know guidelines and standards for GMP, with periodic training of at least 10 days per year (11, 40).

Companies should also make sure that there will be a knowledgeable representative, i.e., Subject Matter Experts (SMEs), available at all times during this process. A checklist at the facility at all times is helpful to ensure everything is updated and working correctly (13).

*Premises and Document Preparation and Presentation*: Inspectors request contract forms with suppliers and third-party acceptors to ensure that outsourced operations meet the required standards (33). They also request documentation to assess integrity, archiving, and storage practices according to guidelines (33) and to follow the ALCOA+ checklist and golden rules of data integrity to ensure that documentation is complete, consistent, and enduring (35).

##### 3.4.2.2 Inspector observations

*Providing Documents Efficiently*: Documents must be easily identified when there is an update, changes in process control, or an increase in the revision number (26).

*Infrastructure Review:* Inspectors examine the company’s infrastructure and facility layout, including aspects such as sanitation, ventilation, pest control, water purity, high-efficiency particulate air (HEPA) filters, heating, ventilation, and air conditioning (HVAC) system, lockers, toilets, and handwashing facilities. Therefore, it is important to prepare paperwork in advance. These are checked against SOPs (32). Inspectors also perform in-process quality control testing, monitor pre-recorded test results (e.g., tablet weight), verify the type of specification for the test, and verify manual and automated processing performance. Inspectors verify that critical manufacturing steps are performed by a responsible individual (batch record, mixing time, sieving, and testing) (32) and use a risk-based approach to assess the implementation of International Council for Harmonisation of Technical Requirements for Pharmaceuticals for Human Use (ICH) guidelines, determining the risk of cross-contamination by evaluating health-based exposure limits (HBEL) (35). Regulations require inspectors to examine in-process materials to ensure conformity with predefined standards, which can be done by sampling and testing (sterility, potency, and contamination control). Yield calculations are also performed to assess the loss in product and how to minimize that loss, so this should also be prepared for (30).

*QMS Review:* Adherence to a robust QMS is a key compliance factor. Companies demonstrate compliance with SOPs, deviation management records, stability and sterility testing, supporting documentation, and supplier oversight processes (1). Inspectors review overall management procedures, including the handling of corrective and preventive actions, (CAPA), and deviations, and evaluate the effectiveness of these systems. Companies are expected to provide past documentation and explain their procedures for handling deviations, as regulatory authorities assess a company’s systematic approach to categorizing deviations against current standards and regulations (29).

Research indicates that inspectors may use a structured questionnaire to evaluate a company’s drug production quality (8). They also inspect packaging, labelling, and finished product quality control testing (32), including the handling of quarantined products awaiting market release (30).

Additionally, computer system validation is reviewed during the inspection. Inspectors assess whether validation steps are documented in line with GMP guidelines and whether installation and operational qualifications are functioning properly. They also check change management logs and ensure all data changes are traceable and compliant (31). Inspectors assess environmental controls for specific procedures and verify whether the equipment is replaced regularly to improve maintenance (29).

##### 3.4.2.3 Responding to inspector observations

*Inspector Interviews and Observations:* Personnel must be aware of all the standard operating procedures. Effective communication is essential, and the CLEAR method should be applied: clarify the question, listen completely, express concisely, avoid speculation, and remain focused (26).

#### 3.4.3 Post-inspection phase (follow-up)

##### 3.4.3.1 Inspection outcomes

Post-inspection follow-up should take place between manufacturers and regulators and is necessary to address findings and ensure compliance (1). All responses to inspectors’ conclusions should be documented for traceability of recorded evidence (33). The inspector may provide suggestions to improve compliance related to staff training (35), changing methodologies (27), enhancing infrastructure, renewing equipment (8), and analyzing workflow (34). GMP inspectors may conduct post-inspection interviews to discuss risk mitigation strategies, using team-based brainstorming to analyze gaps identified during workflow reviews (34).

##### 3.4.3.2 Root cause and CAPA

In cases where inspection results deviate from expected trends, identifying the root cause may require additional time. It will be sent to the company at a later stage of the inspection process. The inspectors may use tools such as the ‘deviation decision tree’ (29) and apply the five-why method to trace the root cause (41). Based on this analysis, CAPAs are recommended to address high-risk areas. Companies can utilize this systematic approach throughout the production process, ensuring lower deviations in future organizational practices (29).

Reporting and follow-up typically occur within 3 weeks of the inspection; a report or a warning letter is sent to the company outlining deficiencies, their classifications, guidance for root cause analysis, and a proposal of a CAPA for each finding (10).

##### 3.4.3.3 Regulatory feedback

Regulatory reports should promote information sharing and should encourage transparency and accountability regarding GMP compliance (36). Regulatory authorities then review company responses (10), and the inspector submits the audit report to the relevant authority (32). For example, the PIC/S council and subcommittee oversee decisions regarding inspection through consensus, using a systematic approach to address issues post-inspection and ensure all participating authorities are aligned (28). Suppose compliance is deemed less than the required standards. In that case, regulatory authorities will refer the company to compliance regulatory groups, and a case management strategy will be developed to guide the company towards regulatory expectations (10).

##### 3.4.3.4 Reacting to the final decision

A final inspection decision is then made, which may include quarantine, approval, or rejection, depending on the region in question (32). Products that pass the inspection are maintained as functional stock under conditions set by the manufacturer (30). Marketing depends on inspection results (32), and if approval is granted, a GMP certificate is issued within a few months (10). Companies are encouraged to analyze regulatory guidance, evaluate post-inspection adherence to in-process and production guidelines, and carry out regular self-inspections for continuous improvement (30). They may also seek support from regulatory authorities or experts for feedback, guidance documents, and SOPs (26, 35). By involving regulators and key stakeholders in post-inspection evaluation, companies can establish a quality culture that supports continuous improvement and stronger management in the market supply chain (5). Quality management personnel would benefit from engaging in discussions about how quality assurance measures can be tailored based on the current inspection report findings for specific products (sterile preparations, biologics, and nanoparticles) and through a retrospective review of reports from previous inspections (1).

##### 3.4.3.5 Adopt a learning and growth mindset

Post-inspection outcomes can provide a unique opportunity for a company’s growth. Companies should learn from previous inspection observations made by authorities and ensure that these have been addressed. A specific action plan must be followed. A written report outlining deficiencies will be sent to the site generally within 3 weeks of the last day of the inspection. The site is asked to respond to these deficiencies. The primary report will specify the deadline for this response. Before addressing the report, a designated response team should be established (10).

Every deficiency should have an actionable response and a set timeline. A detailed root cause analysis and CAPA suggestion should be provided. Ensuring that, if applicable, a wider review is conducted to confirm there are no similar issues in other related areas. Having a standard investigation template for root cause analysis is important. It is expected that this response will specify particular activities, including the actions to be taken, the responsible individuals, and the completion timeframe.

After observations from an inspection are noted, effective change management may be needed in accordance with the site change control procedure. Change management involves overseeing changes in staff, protocols, operations, and product standards. It is part of GMP compliance because regulatory agencies require companies to demonstrate their ability to handle change. It must be ensured that the observations are communicated around the manufacturing network so that other sites that may share the same company procedures do not encounter the same issue (27).

The inspection will be closed, and authorities will send a close-out letter and GMP certificate once the response to the inspection report is deemed acceptable (13).

## 4 Discussion

**Figure 3:**
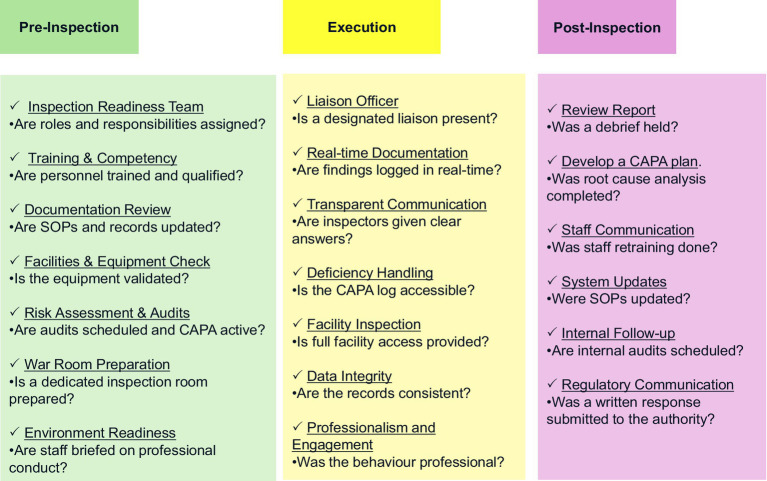
GMP inspection readiness and checklist model.

This scoping review mapped existing literature on GMP inspection management. It incorporated a mix of peer-reviewed academic sources and grey literature, including regulatory guidelines (such as those from the WHO and the HPRA), industry reports, empirical studies, and other resources. Thematic analysis identified key GMP factors that influence inspection outcomes. It examined how companies can prepare for inspections, execute them, and arrange post-inspection activities. This review differs from existing regulatory frameworks, such as those issued by the EMA, the FDA, and the WHO, because it consolidates practical inspection management strategies across the pre-, during-, and post-inspection phases from academic and grey literature sources. Whereas existing frameworks mainly provide compliance requirements, this review uniquely highlights how companies can operationalize regulatory expectations through a checklist model (see Figure 3). Therefore, the study bridges the gap between regulatory theory and real-world inspection execution.

The majority of the findings were derived from the academic literature; however, this may not accurately reflect inspection practices within Europe, as there was a surprising scarcity of peer-reviewed studies that are based in that region.

### 4.1 Weak regulatory enforcement

Thematic analysis identified several challenges and regional disparities. In developing countries such as India and China, weak regulatory enforcement and limited inspector capacity contribute to inconsistent GMP implementation. Such countries lack a robust framework for navigating GMP, which increases the risk of lower product quality standards or inaccurate inspection outcomes. In contrast, developed regions such as the EU and the US benefit from harmonized inspection systems, more frequent training, and structured data review processes. However, even among advanced regulatory bodies, research showed a lack of peer-reviewed discussions and transparency (36).

### 4.2 Organizational size

Small- and medium-sized enterprises (SMEs) dominate developing markets (98% of total firms) (8). Compared to multinational manufacturers operating within mature systems, such as the EMA, these companies face challenges in accessing guidance on GMP implementation (34). The demand for resources to meet rising quality standards creates economic challenges for companies, particularly SMEs, adding financial burdens (8). However, cost–benefit considerations for specific processes, such as ICH-Q3D (42) or GDocP (33), are not widely explored in the literature. There are also fewer discussions on the failure of certain processes, such as the failure of CAPA implementation and its implications for manufacturers (29). This lack of transparency affects not only SMEs but also regulators, who are estimating the cost of on-site inspections or aiming to join global networks such as PIC/S. The cost–benefit analysis of joining these networks is not discussed compared to joining other local GMP frameworks (28). Guidance should be equally accessible (34), and biased support towards larger companies should be avoided (30).

### 4.3 Personnel

Long-term investment in quality personnel is important for GMP inspection preparation and compliance. Companies should promote a culture of quality awareness and improve management strategies (8), as research has shown that gaps and ambiguities in manufacturers’ understanding of what constitutes substandard products can lead to regulatory approval delays (36). Companies should be encouraged to conduct anonymous employee surveys using open-ended questions to gather feedback on GMP compliance respectfully and confidentially (5).

### 4.4 Data sharing

Studies also show that companies are hesitant to share product rejection metrics. To address this, companies should consider anonymously sharing internal quality data to enhance inspection readiness and harmonization (5). Similarly, researchers face challenges accessing GMP inspection results due to the absence of professional data collection systems, leading to a lack of empirical research and negatively impacting the pharmaceutical industry worldwide. Reporting post-inspection consequences and penalties issued by regulatory bodies enforces accountability and encourages transparency and learning in the industry to prevent future violations (8). Mutual recognition agreements between central regulatory authorities (such as the FDA and EMA) allow for reliance on one another’s GMP inspections (43). However, differences in how GMP standards are interpreted limit harmonization (36). These disparities across companies highlight the need for a more structured and standardized approach to quality assurance across the pharmaceutical industry (8).

### 4.5 Regulatory changes, data integrity, and future directions

Constant updates to GMP guidelines pose challenges to inspection readiness, requiring companies to adapt to meet high standards continuously. One notable risk area involves transitioning from paper-based to electronic systems, which can affect data storage, traceability, and transcription integrity, especially in remote or hybrid inspections (11, 35). A solution to this is the utilization of an electronic quality management system (QMS) that streamlines the documentation process to reduce human errors and improve traceability (6). Data integrity remains a critical concern, with issues such as test result manipulation or falsification of quality control logs compromising the reliability of GMP inspections (29). Emerging technologies offer promising solutions. Blockchain technology networks, for instance, can detect unauthorized alterations and safeguard data integrity, thereby strengthening data authenticity and transparency (14, 42). Similarly, artificial intelligence (AI) tools are increasingly being used in inspection planning to prioritize high-risk areas, detect patterns of non-compliance, and proactively address issues that could lead to drug shortages and unmet patient needs (1, 14, 44). These digital innovations represent important steps towards modernizing inspection practices and improving regulatory efficiency globally.

### 4.6 Unknown unknowns

Donald Rumsfeld’s well-known quote on ‘unknown unknowns’ can be relevant as pharmaceutical companies approach the pre-inspection phase, highlighting the risk of unrecognized gaps. As Rumsfeld, then Secretary of Defence, said in 2002, ‘There are known knowns; there are things we know we know. We also know there are known unknowns; that is to say, we know there are some things we do not know. However, there are also unknown unknowns, the ones we do not know we do not know’. Pharmaceutical companies often enter a GMP inspection aware of specific compliance requirements, i.e., known knowns. The most significant red flags, meanwhile, come from the ‘unknown unknowns’: problems they do not know exist until inspectors call attention to them, such as a company not having an ongoing audit program with their suppliers. Proper inspection preparation, execution leadership, and follow-up can help to manage known knowns and unknown unknowns (45).

### 4.7 Strengths and limitations

The risk-of-bias assessment was conducted by a single reviewer, which may limit the reliability of the results and increase the likelihood of selection bias. However, explicit inclusion and exclusion criteria, use of the MMAT tool, and secondary verification and revisions minimized this risk and revealed notable trends.

Grey literature was a valuable component for our review, as it provided a broader scope of evidence by including government resources and industry perspectives. However, it lacks the rigorous peer-reviewed process characteristic of academic resources, making it more prone to bias, errors, and selective reporting. Unlike academic literature that goes through continuous revisions through citation and follow-up research, grey literature usually lacks methodological scrutiny.

This study produced GMP inspection checklists that companies can use to guide them through the inspection process, and it provides a globally relevant perspective on GMP compliance, challenges, and inspection practices, which can be developed further.

## 5 Conclusion

Our evidence synthesis for this scoping review of good manufacturing practice inspection management indicates a scarcity of qualitative research in this field. This study analyzed preparation, execution, and post-inspection practices, identifying key challenges, ethical concerns, and opportunities for improvement. The findings have developed a framework for GMP inspection management and highlight key aspects, including documentation practices, personnel training, internal audits, and regulatory elements such as inspectors’ training and selection for a facility. Post-inspection practices, including CAPA implementation and explanation of the inspection outcome, were also explored.

This study also highlights the role of grey literature in exploring the breadth of GMP inspection management while acknowledging its limitations.

## Data Availability

The original contributions presented in the study are included in the article/supplementary material, further inquiries can be directed to the corresponding author.
