# Supplementary material for: The management of good manufacturing practice (GMP) inspections: a scoping review of the evidence
Source: Front Med (Lausanne). 2025 Nov 11;12:1687864. doi: 10.3389/fmed.2025.1687864 (PMC12645793; doi:10.3389/fmed.2025.1687864)
Supplement: Supplementary file 1 [file Data_Sheet_1.docx]

**Supplementary File 1**

**Table 1:**

**Academic literature search strategy [Search date, Database, Search string, Results]**

|  | Date | Database | Search String copy and paste | Results in figures |
| --- | --- | --- | --- | --- |
| 1 | 10/02/2025 | Embase | (pharmaceutical:ab,ti OR drug:ab,ti OR 'medicinal product':ab,ti OR 'licensed product':ab,ti OR medicine:ab,ti) AND english:la AND [2015-2025]/py | 1,459,017 |
| 2 | 10/02/2025 | Embase | ('good manufacturing practice':ab,ti OR gmp:ab,ti) AND english:la AND [2015-2025]/py | 11,156 |
| 3 | 10/02/2025 | Embase | inspection:ab,ti OR quality:ab,ti OR 'gmp inspection':ab,ti OR 'pharmaceutical inspection':ab,ti OR 'regulatory inspection':ab,ti OR 'gmp audit':ab,ti OR review:ab,ti AND english:la  AND [2015-2025]/py | 2,693,951 |
| 4 | 10/02/2025 | Embase | (management:ab,ti OR 'best practice':ab,ti OR 'technical factors':ab,ti OR 'management aspect':ab,ti OR 'social factor':ab,ti OR lead*:ab,ti OR organis*:ab,ti) AND english:la  AND [2015-2025]/py | 2,801,151 |
| 5 | 10/02/2025 | Embase | ('pharmaceutical industry':ab,ti OR pharma:ab,ti OR manufacturing:ab,ti) AND english:la AND [2015-2025]/py | 79,883 |
| 6 | 10/02/2025 | Embase | 1+2+3+4+5 | 169 |
| 7 | 10/02/2025 | Citation search | 1 | 1 |
| 8 | 10/02/2025 | PubMed | Pharmaceutical[Title/Abstract] OR drug[Title/Abstract] OR medicinal product[Title/Abstract] OR licensed product[Title/Abstract] OR medicine[Title/Abstract] Filters: English, from 2015 – 2025 | 1,130,231 |
| 9 | 10/02/2025 | PubMed | Good manufacturing practice[Title/Abstract] OR GMP[Title/Abstract] Filters: Free full text, Full text, English, from 2015 – 2025 | 7,411 |
| 10 | 10/02/2025 | PubMed | Inspection[Title/Abstract] OR Quality[Title/Abstract] OR GMP inspection[Title/Abstract] OR Pharmaceutical inspection[Title/Abstract] OR regulatory inspection[Title/Abstract] OR GMP audit[Title/Abstract] OR review[Title/Abstract] Filters: English, from 2015 – 2025 | 2,207,010 |
| 11 | 10/02/2025 | PubMed | Management[Title/Abstract]OR Best Practice[Title/Abstract] OR technical factors[Title/Abstract] OR management aspect[Title/Abstract] OR social factor[Title/Abstract] OR lead*[Title/Abstract] OR organis*[Title/Abstract] Filters: English, from 2015 – 2025 | 2,124,336 |
| 12 | 10/02/2025 | PubMed | Pharmaceutical industry[Title/Abstract] OR pharma[Title/Abstract] OR manufacturing[Title/Abstract]  Filters applied: English, from 2015-2025 | 66,166 |
| 13 | 010/02/2025 | PubMed | 8+9+10+11+12+13 | 88 |

| Title of study  **Table 2: characteristics of included studies** | Author’s  Name(s) | Year /Country | Study design | Data collection method | Data analysis | Aim of study | Reported outcome | Limitation | Funding |
| --- | --- | --- | --- | --- | --- | --- | --- | --- | --- |
| Quality Assurance: Role in the Pharmaceutical Industry (36). | Suruchi Agarwal. | 2023, India. | Review article. | Secondary data from research papers, regulatory guidelines, industry reports, case studies. | Thematic analysis of quality assurance principles, elements, and regulatory compliance in the pharmaceutical industry. | To highlight the role of quality assurance in pharmaceutical manufacturing, emphasizing good manufacturing practices, regulatory compliance, and quality control measures. | Importance of QA in pharmaceuticals, key components of QA, challenges in QA, recommendations in QA. | N/A | Not declared. |
| Change Control from Initiation to Implementation in the Pharmaceutical Industry: A Comprehensive Review (33). | Soaib Ahmed, Souvik Kundu, Rahul Patra, Jaydip Ray. | 2024, India. | Comprehensive Review. | N/A | Qualitative narrative review. | outlines change control management in the pharmaceutical industry, emphasising regulatory compliance, risk assessment, and systematic implementation of changes. | systematic control of change to ensure product quality. | A review study. | No funding was given. |
| Pharmaceutical Inspection Co-operation Scheme: An Overview (43). | Prasanna Gayathri B, Kamaraj R. | 2024, India. | Review article. | qualitative research / observation. | qualitative analysis approach. | This study's purpose is to establish uniform GMP standards by providing extensive training for inspectors and promoting collaboration and networking among regional and international bodies, regulatory authorities, and other relevant organisations. | Importance of compliance, strategic development, PIC/S benefits for members and industry, audit checklist summary. | N/A | No funding was received. |
| Risk assessment of GMP inspection of overseas pharmaceutical manufacturers based on desktop inspection as required in Thailand (49). | Varin Titapiwatanakun, Waranon Cheewajorn, Narueporn Sutanthavibul. | 2023, Thailand. | This work utilized the QRM in ICH guideline Q9 and FMEA tool to evaluate the GMP desktop inspection system in the scope of Overseas Pharmaceutical Manufacturers by carrying out five steps: (1) Pre-assessment, (2) risk identification, (3) risk analysis, (4) risk evaluation, and (5) risk reduction. | Risk identification and analysis were performed by setting up an interdisciplinary team, collecting statistical data of the GMP desktop situation and drug quality defects. | Failure Mode and Effects Analysis (FMEA) was used to evaluate the risks based on the scores of occurrence, severity, detectability and RPN. Thai regulations were compared with international standards to identify regulation gaps and workflows. | The study aimed to assess the gaps in terms of quality and reliability associated with the GMP inspection of overseas pharmaceutical manufacturers in Thailand based on desktop inspections, particularly when compared with on-site inspection. | The results showed that all of the new risk priority number changed to an acceptable level. | FMEA can introduce bias in scoring risk factors since it relies on expert judgment. The study only focused on Thailand's regulatory framework GMP inspection data was collected from 2016 to 2018, which may not reflect current regulatory regulations. | No funding was declared. |
| Characteristics, risk management and GMP standards of pharmaceutical companies in China (11). | Hong Chen, Lijian Qin, Cong Jiang, Mingshuai Qin, Yanming Sun and Jingjing Luo. | 2023, China. | Quantitative analysis using the 2SLS regression model based on the real GMP inspection results from the Chinese National Medical Products Administration. | On-site GMP inspection results in China. | A 2SLS regression model was used to investigate which factors affect GMP inspection outcomes. Simultaneously, the Multinomial Logit Model, the Ordered Logit and Ordered Probit model were used to test the robustness of the measurement results The logarithm of the one- year lag of value of fixed assets was taken to eliminate the non-normality of the data. | The study aimed to enable initiate an empirical analysis of how company characteristics and risk management affect the GMP inspection results of certain pharmaceutical companies. | First, compared with Chinese state-owned companies, foreign commercial and private enterprises are held to higher standards. Second, the GMP inspection results tend to be better for those enterprises whose main sources of capital are not dependent on bank loans. Third, enterprises with higher fixed assets tend to receive the better GMP inspection results. Fourth, the longer the quality authorized staff has worked in a company, the better the GMP inspection results expected of that enterprise. | The number of sample companies has to be increased. In the existing literature study in this field, the sample amount used herein is relatively, but the representation of the sample can be further enhanced if the number of observed values of the existing samples can be largely expanded. Second, the sample uses cross-sectional data and cannot eliminate the influencing factors that do not change over time. | Social science foundation of Anhui province of China -key project of humanity and social science supported by the bureau of education of Anhui province - major project of the social science foundation of Anhui province of China. |
| Deviations handling and corrective actions and preventive actions: Case studies for parenteral dosage form (29). | Shah Kavina, Kothari Charmy, Modi Chirag and Shah Manan. | 2019,India. | Review paper. | Primary data: analysis of real case studies regarding deviations in parenteral dosage manufacturing and CAPA application Secondary data: literature review on GMP and QMS, regulatory requirements, and industry best practices about risk assessment, CAPA and deviation. | N/A | Deviations have to be addressed, investigated, and based on investigation data, suitable corrective actions and preventive actions are applied for prevention of its reoccurrence. | Deviations were inevitable in pharmaceutical manufacturing due to equipment or instrument failures, human errors, or environmental conditions. CAPA proactively improved manufacturing and regulatory compliance as case studies demonstrated. | N/A | No funding was declared |
| Analysis of non-compliances identified in GMP inspections between 2013 and 2022 (2). | Hristina Lebanova, Svetoslav Stoev, Galina Petrova. | 2024, Bulgaria. | A retrospective analysis of non-compliance reports published in the Eu draGMDP database between 2013 and 2022 was performed. | The data in EudraGMDP The analysis of the 99 reports published in EudraGMDP by national competent authorities on non-compliant operations identified during inspections carried out between January 2013 and December 2022 shows that non-conformities with GMP rules were identified in manufacturers in 19 countries on 5 continents. | The study analysed: The number of non-compliances identified during GMP inspections for the period 2013–2022. • Type of non-compliance and actions taken. Data was analysed using descriptive statistics. Initial data processing, entry and subsequent analysis were performed using Microsoft Excel 2019. For the summary of quantitative parameters, the number of responses, mean, median, standard deviation, minimum and maximum values were presented. | The aim of the present study is to analyse the non-compliant operations identified during GMP inspections carried out by national competent authorities (NCA) in the EU/EEC between 2013 and 2022. | The observed deficiencies highlight the importance and need for continuous monitoring and improvement of manufacturers’ production processes and quality management systems. | COVID 19 impact in data. | Funding was not declared. |
| Incentivizing Quality in the Manufacture of Pharmaceuticals: Industry Views on Quality Metrics and Ratings (7). | Cesar Medina, Frances J. Richmond. | 2015,USA. | A survey-based research design. | A 23-question survey engaged 2 separate populations of industry professionals: a panel of identified US industry experts (n = 110) and a broader population (n = 328) of professionals working primarily in southern California. | Descriptive statistics: mean values, standard deviations, and response frequencies were calculated to evaluate 1) Metrics commonly used to assess company and product quality 2)Metrics that companies might be willing to share anonymously for industry quality improvement efforts 3)Level of agreement on how quality valuations might be influenced by quality ratings 4)Impact that quality ratings may have on company decisions. | The study aimed to investigate industry views related to the use of quality metrics and ratings to investigate how quality metrics influence regulatory inspections, incentivize higher quality manufacturing and affect stakeholder decision-making. | Industries valued FDA warning letters and other observations the most. Respondents were generally hesitant to share information regarding process performance measures that may warn of problems. The expert panel and open-access groups indicated a high level of agreement and a high level of congruence on the effect that quality scorecards might have and suggested that the quality ratings might be particularly influential on manufacturers. Both the EP and OA respondents predicted that the strongest effects would be seen on internal and supplier quality audits, employee training, and the emphasis on quality as a more company strategic goal. | Additional research is needed to understand how patients may use quality information to decide what medical products are best for them. It should gain understanding of how wholesalers and group purchasing organizations can use quality information. Sample bias can be seen due to small size of expert panels, highly specialized group, and respondents were mainly from Southern California. | No financial support was given. |
| Regulatory Comparative Quality Systems of India and USA and its Significance in Pharmaceutical Industry Facilities (40). | Prabhakar V S Beula Evangeline C Aravindhanathan V Sruthi N Gowthamarajan K. | 2020, India. | Comparative Analysis. | Literature review. | Qualitative Comparative analysis. | The aim of this research paper is to compare the regulatory requirements of USFDA and CDSCO-Schedule M in GMP, focusing on their impact on pharmaceutical enforcement and regulatory approval in the U.S. and India. It examines challenges faced by pharmaceutical companies, proposes solutions for GMP compliance, and evaluates the effectiveness of quality management systems through regulatory inspections. The study also differentiates application types that trigger inspections and analyses their outcomes. | The study concludes that a standardized approach is needed for preparing a single site master file, as both Schedule M and USFDA outline quality management requirements under different headings. While both regulations provide specifics on quality control, GMP remains essential for ensuring product quality through proper manufacturing and testing practices. | Limited scope- The study focuses only on USFDA and CDSCO-Schedule M regulations, excluding other global regulatory frameworks like EMA (Europe) or WHO GMP guidelines. | No support was given |
| The Essential Guide to Computer System Validation in the Pharmaceutical Industry (37). | Jawahar Rohith Raja Alekhya Kella Damodharan Narayanasamy. | 2024, India. | Review article. | Literature review. | Comparative analysis of various validation methods and regulatory guidelines, with practical examples and case studies. | The study provides a guide to computer system validation (CSV) in the pharmaceutical industry, ensuring systems produce reliable data and meet regulatory requirements. CSV covers the entire system lifecycle, from planning to modification, with validation steps to ensure compliance. | The study highlights the benefits of CSV, including improved data assurance, reduced validation costs and time, increased compliance with GMP and 21 CFR Part 11 regulations, and enhanced product quality and safety. It also outlines the steps involved in the CSV process, from planning to periodic review. | It is a review article and does not present original experimental data. | No financial support was received. |
| Auditing on regulatory six system in pharmaceutical industry, a review (41). | P. Shanmugapriya, R. Sanilkumar. | 2019, India. | Review article. | Document review. | Qualitative descriptive approach. | Explain six system auditing models and the guidelines for regulatory inspectors. | How auditing should be held - six system inspection. | This is a review article. | No funding was declared. |
| Recent Regulatory Trends in Pharmaceutical Manufacturing and their Impact on the Industry (35). | Daniel Tabersky Michael Woelfle Juan-Antonio Ruess Simon Brem Stephan Brombacher. | 2018, Switzerland. | Review article. | Analysis of recent guidelines and regulations in the pharmaceutical industry/ document review. | Comparative analysis of the impact of new guidelines on the industry, with specific examples. | To highlight the impact of recent regulatory changes on the pharmaceutical industry, focusing on three specific guidelines: ICH Q3D for elemental impurities, EU-GMP Guideline Part III Chapter on health-based exposure limits, and PIC/S 041-1 on data integrity. | The study discusses how these guidelines have modernized control strategies, improved product quality, and increased patient safety. It also emphasizes the importance of data integrity and the scientific approaches used to comply with these regulations. | The article primarily focuses on the regulatory environment in Switzerland and may not cover all global regulatory trends. Additionally, it is a review article and does not present original experimental data. | No funding was declared. |
| Institutional barriers and enablers to implementing and complying with internationally accepted quality standards in the local pharmaceutical industry of Pakistan: a qualitative study (1). | Fatima Tauqeer, Kirsten Myhr, Unni Gopinathan. | 2019, Pakistan. | Qualitative case study involving 22 interviews of the drug regulatory bodies (n=9), academia (n=3) and local manufacturers (n=10), identifying key themes in data by thematic analysis. | A qualitative case study design. | Document analysis was used to collect additional information and supplement the interview data. The aims of the qualitative data analysis were: (1) to identify common themes from across the three main stakeholders; and (2) to identify contrasting experiences and views on the topics raised across the three main stakeholders. | The study investigated: (1) How is quality assurance (QA) and GMP compliance understood and acted upon by local pharmaceutical manufacturers; (2) What are the institutional barriers and enablers for QA and GMP compliance in the local pharmaceutical sector from the perspective of key stakeholders; and (3) What are the institutional barriers and enablers for strengthening local regulatory capacity to improve QA in the industry in the long term. | There is lack of harmonization in quality standards across manufacturing facilities. GMP compliance remains in consistent with only few companies achieving high-quality standards Finance constraints, infrastructure issues, and shortage of skilled workforce are barriers to maintain sustainable GMP compliance. Manufacturers prioritize profit over quality. Drug Regulatory Authority of Pakistan lacks capacity, resources, and enforcement Pakistan joined WHO's Collaborative Registration Procedure to enable for improvement of GMP compliance. | The data was sourced from a limited number of companies from one geographic location (Lahore) - may not represent general landscape. Potential selection bias. | -Travel funding by the institute of health and society, university of Oslo  -Research council of Norway’s global health and vaccination program. |
| A Comprehensive Review on Comparison of GDocP in Pharmaceutical Manufacturing Unit as per European and WHO Guidelines (34). | Male Tejaswi Vinod Kumar K. Chithra Shekar C. Srikanth K. | 2021, India. | Review article. | Literature review of European and WHO guidelines. | Comparative analysis of European and WHO guidelines on Good Documentation Practices (GDocP). | To provide an overview and comparison of Good Documentation Practices (GDocP) in pharmaceutical manufacturing as per European and WHO guidelines. | Both European and WHO guidelines have similar principles, with WHO guidelines being more detailed in some aspects. ALCOA and ALCOA+ principles were emphasized as key to maintaining data integrity. | does not discuss other regulatory frameworks (include only WHO and EU guidelines). | No funding was received for this study. |

**Table 3 Mixed method appraisal tool (MMAT 2018) Quality appraisal**

|  | Title of study | Screening questions | | Criteria 1 | Criteria 2 | Criteria 3 | Criteria 4 | Criteria 5 | Total score  (based on the five criterion) | Quality of study |
| --- | --- | --- | --- | --- | --- | --- | --- | --- | --- | --- |
|  |  | **S1** | **S2** |  |  |  |  |  |  |  |
| Study 1 | Quality Assurance: Role in the Pharmaceutical Industry (36). | No, does not specify a clear research question. | Cannot tell, study rely mainly on review of existing literature. | ----- | ----- | ----- | ----- | ----- | ----- | ----- |
| Study 2 | Change Control from Initiation to Implementation in the Pharmaceutical Industry: A Comprehensive Review (33). | Yes, but it is implied rather than explicitly stated. | Yes, data comes from regulatory guidelines, and industry standards. | Cannot tell, since it relies mostly on secondary sources of existing information. | Cannot tell, the author interpret data from secondary sources to deduce his conclusions. | Yes, the findings are derived from existing secondary sources. | Yes, excerpts and citations from regulatory guidelines is provided to support data collection and help with analysis. | No, there is clear links between data source, collection, analysis, and interpretation, however it does not include qualitative data collection. | 2/5 | weak quality. |
| Study 3 | Pharmaceutical Inspection Co-operation Scheme: An Overview (43). | Yes, but it is implied rather than explicitly stated. | Yes, mainly from secondary sources to provide a broad policy analysis. | Yes, by analysing policy guidelines to answer the questions using qualitative and observational approach. | Cannot tell, data collection method used to answer the research questions is mainly document-based. | Cannot tell, due to the element of observational data analysis, we cannot tell if the analysis is based on author’s interpretation or deduced from collected data. | No, this study allow for thematic justifications via regulations and not quotes. | Yes, there is clear links between data source, collection, analysis, and interpretation. | 2/4 | weak quality. |
| Study 4 | Risk assessment of GMP inspection of overseas pharmaceutical manufacturers based on desktop inspection as required in Thailand (49). | Yes, the study have a clear research questions that is addressed along the way. | Yes, documentation gathered used to addressed those questions. | Cannot tell, we cannot confirm appropriateness of the qualitative approach. | Yes, various data collection methods were utilised for this study (documents, interviews).. | Yes, findings was derived from the data and represented in a graph and risk priority number was calculated. | Cannot tell, the study does not give adequate quotes to justify the themes. | Yes, risk analysis is derived from collected data sources, which provided coherence to the study. | 3/5 | Moderate quality. |
| Study 5 | Characteristics, risk management and GMP standards of pharmaceutical companies in China (11). | Yes, a research question is clearly identified. | Yes, it is based on actual GMP inspections results that were gathered over three years. | No, it is mainly based on quantitative methods such as 2SLS regression to analyse results of GMP inspections. | No, it depends mainly on company inspections and financial reports. | Yes, it uses 2SLS regression and descriptive statistics for further support of the findings. | Yes, descriptive statistics was employed for further interpretation of the analysis. | Cannot tell, the study was mainly quantitative in nature. | 2/5 | Weak quality.  (potential for bias due to government funding nature of this study, better bias analysis is needed for this study) |
| Study 6 | Deviations handling and corrective actions and preventive actions: Case studies for parenteral dosage form (29). | Yes, but is rather implied than explicitly stated. | Yes, by showing case studies of deviations. | No, it does not use purely qualitative data collection methodology and mainly descriptive in nature and relies on case-studies. | Cannot tell, because it relies mainly on case studies and deviations and is unclear about the observatory approach in the study . | Yes, it shows deviation handling based on case studies. | Yes, preventative action plan was formulated according to provided case studies. | No, it does not focus on qualitative data analysis such as interviews and thus does not follow the structure of qualitative analysis. | 2/5 | Weak quality |
| Study 7 | Analysis of non-compliances identified in GMP inspections between 2013 and 2022 (2). | Yes, research questions are defined. | Yes, it include inspections reports from different regulatory authorities to identify non-compliance. | No, the study uses mainly quantitative methods such as inspections reports and descriptive statistics rather than qualitative approach such as interviews. | No, it relies on retrospective analysis of structured GMP report, and not qualitative data collection method. | Yes, the analysis of inspection reports leads to identifying and categorizing deficiencies. | Yes, it supports it with quantitative analysis approach using figures and tables. | Cannot tell, this study is not of qualitative nature mainly. | 2/5 | Weak quality. |
| Study 8 | Incentivizing Quality in the Manufacture of Pharmaceuticals: Industry Views on Quality Metrics and Ratings (7). | Yes, research questions are clear. | Yes, quantitative and qualitative approaches were used to address the research questions. | Yes, it used a qualitative approach by employing surveys. | Yes, surveys were adequate to address the research questions, it contained open ended questions. | Yes, the responses gathered from the survey were analysed and quantitative analysis (descriptive statistics) was used to further support the findings. | Yes, statistical and descriptive analysis were used to interpret the data. | Cannot tell, the study was not clear on connecting all elements even though it used both qualitative and quantitative approach. | 4/5 | Good quality. |
| Study 9 | Regulatory Comparative Quality Systems of India and USA and its Significance in Pharmaceutical Industry Facilities (40). | Yes, there are clear research questions. | Yes, the collected data (secondary data sources) address the research questions. | Yes, the study uses observational qualitative method approach. | Cannot tell, even though it has an qualitative observational approach, it relied mostly on secondary sources such as regulatory guidelines. | Yes, findings were deduced from collected data of secondary sources. | Yes, it is supported by comparison between regulatory guidelines. | Cannot tell, due to the weakness of the qualitative element in this study. | 3/5 | Moderate quality. |
| Study 10 | The Essential Guide to Computer System Validation in the Pharmaceutical Industry (37). | Yes, however it is implied and not explicitly stated. | Yes, the data addresses the research question. | Yes, Descriptive observational review is given and has a qualitative approach to it by focusing on explaining concepts rather than statistics. | Cannot tell, it uses secondary data to deduce an analysis. | Yes, the findings are based on collected secondary data. | Cannot tell, it does not mention real life CSV implementation which makes it hard to determine data interpretation results. | Cannot tell, it is unclear how data was selected and this leads to undefined coherence in this study. | 2/5 | Weak quality. |
| Study 11 | Auditing on regulatory sic system in pharmaceutical industry: a review (41). | Yes, however it is implied and not explicitly stated. | Yes, the data address the research questions. | Yes, it uses document review and has a descriptive qualitative element to it. | Cannot tell, the study uses secondary data and analyse it using qualitative approach however it does not use interviews, surveys or other qualitative methods. | Yes, it is derived from gathered secondary sources. | No, the author drew interpretations from existing regulatory guidelines and not from new collected data. | Cannot tell, since the qualitative element is weak and the main focus is on secondary data. | 2/5 | Weak quality. |
| Study 12 | Recent Regulatory Trends in Pharmaceutical Manufacturing and their Impact on the Industry (35). | Yes, however it is implied and not explicitly stated. | Yes, the data address the research questions. | Yes, it is appropriate by qualitative analyses of regulatory changes , it also uses documents reviews. | No, the study relies on document review and secondary data for data collection but the analysis is qualitative in nature. | Yes, it is derived from regulatory updates and other secondary sources. | Yes, it provided a good regulatory analysis and implementation of the guidelines. | Cannot tell, since the data collection method wasn’t qualitative in nature so it lacked the qualitative structure. | 3/5 | Moderate quality. |
| Study 13 | Institutional barriers and enablers to implementing and complying with internationally accepted quality standards in the local pharmaceutical industry of Pakistan: a qualitative study (1). | Yes, there is explicit research questions. | Yes, the documents and the interviews collected sufficient data to answer the question. | Yes, it uses qualitative based study design (semi-structured interviews). | Yes, the qualitative data collection method was adequate to address the question. | Yes, the findings were derived from collected data. | Yes, it provided stakeholder’s quotes which further supported interpretation of data. | Yes, it provided coherence throughout the study. | 5/5 | Excellent quality. |
| Study 14 | A Comprehensive Review on Comparison of GDocP in Pharmaceutical Manufacturing Unit as per European and WHO Guidelines (34). | Yes, the article have a clear research questions. | Yes, it addressed research questions with information from appropriate data sources. | Cannot tell, the study compares GDocP based on EU and WHO guidelines uses document reviews and secondary data analysis. | Yes, data collection method was appropriate to address the research question. | Yes, the findings are linked to the guidelines and secondary data collected for analysis. | Yes, the study cite GMP guidelines and is sufficient in interpretation of data. | Cannot tell, the study is a review and does not involve the coherence qualitative structure. | 3/5 | Moderate quality. |

Screening questions :

S1. Are there clear research questions?

S2. Do the collected data allow to address the research questions?

Further appraisal is not appropriate if one or two of the answers to the screening questions is ‘No’ or ‘Cannot tell’.


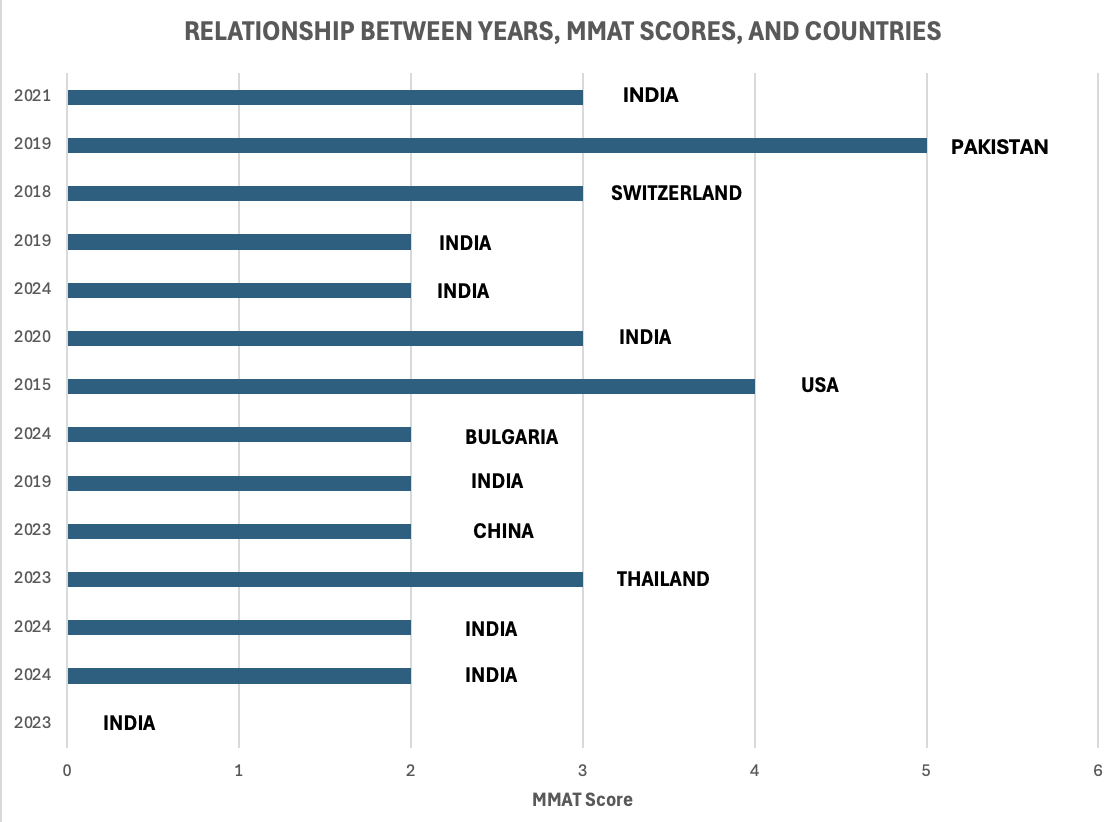


GRAPH 1 Relationship between years, MMAT scores, and countries.

**List of abbreviations**

| Abbreviation | Full Term |
| --- | --- |
| AI | Artificial Intelligence |
| ALCOA+ | Attributable, Legible, Contemporaneous, Original, Accurate + Complete, Consistent, Enduring, and Available |
| CAPA | Corrective and Preventive Action |
| CSV | Computer system validation |
| EMA | European Medicines Agency |
| EU | European Union |
| FMEA | Failure Mode and Effects Analysis |
| FPQC | Finished Product Quality Control |
| GDocP | Good Documentation Practice |
| GMP | Good Manufacturing Practice |
| HBEL | Health-Based Exposure Limit |
| HVAC | Heating, Ventilation, and Air Conditioning |
| ICH | International Council for Harmonisation of Technical Requirements for Pharmaceuticals for Human Use |
| ICH Q3D | Guideline for Elemental Impurities |
| MRA | Mutual Recognition Agreement |
| MMAT | Mixed Methods Appraisal Tool |
| PIC/S | Pharmaceutical Inspection Co-operation Scheme |
| PDE | Permitted Daily Exposure |
| PRISMA-ScR | Preferred Reporting Items for Systematic Reviews and Meta-Analyses extension for Scoping Reviews |
| QMS | Quality Management System |
| RPN | Risk Priority Number |
| SOP | Standard Operating Procedure |
| SPIDER | Sample, Phenomenon of Interest, Design, Evaluation, Research type |
| SMEs | Small and medium sized enterprises |
| WHO | World Health Organization |
