# Supplementary material for: The management of good manufacturing practice (GMP) inspections: a scoping review of the evidence
Source: Front Med (Lausanne). 2025 Nov 11;12:1687864. doi: 10.3389/fmed.2025.1687864 (PMC12645793; doi:10.3389/fmed.2025.1687864)
Supplement: Supplementary file 2 [file Data_Sheet_2.docx]

**Supplementary File 2**

**Table 4 Grey Literature Database Search strategy (19)**

1-Search for a variety of word combinations.

- Search terms for all: Pharmaceutical, Good Manufacturing Practice, Inspection, Management, Pharmaceutical industry
- Search engine: Google Advanced, applying filters for English only, searching for the exact word or phrase “good manufacturing practice” and any of these words: management, best practice, drug or GMP audit

2- Determine how items will be selected for screening (189 items), import them into a shared document, and divide them among four people for the first screening, second screening, and consensus by a third reviewer.

3- Search conducted **17/02/2025**

4- Data extraction for grey literature was performed by one reviewer and is shown below

|  | **URL** | **1st screening** | **2nd screening** | **Consensus** | **Organisation name** | **Year of publication/last update** | **Country/ies** | **Aim /objectives** | **Outcome** | **Types of Sources** | |
| --- | --- | --- | --- | --- | --- | --- | --- | --- | --- | --- | --- |
| 1 | <https://www.ema.europa.eu/en/human-regulatory-overview/research-development/compliance-research-development/good-manufacturing-practice> | Exclude | Include - | **Include** | European medicine agency (EMA) | Year of publication: N/A  last update: N/A  multiple files | Europe | -identify EMA standards to compare to other regulators.  -inspectors responsibility. | -establishing EMA role in inspections. | Regulatory authority | |
| 2 | <https://www.fda.gov/drugs/pharmaceutical-quality-resources/facts-about-current-good-manufacturing-practice-cgmp> | Exclude | Include | **Include** | Food and drug administration (FDA) | Year of publication: N/A  last update: 2025 | USA | -understand requirements of cGMP.  -who is responsible for manufactures inspection.  -an advice to the public when consuming  medication from a non-GMP compliant manufacture. | -balancing risk-benefit when healthcare practitioners choose to prescribe medications from a non-GMP compliant manufacturer  -how the public is protected when GMP is violated.  -a guideline to new companies of what is required to cGMP. | Regulatory authority | |
| 3 | <https://www.hpra.ie/regulation/human-medicine/manufacturers/good-manufacturing-practice-(gmp)-inspections> | Include | Include | **Include** | Health product regulatory authority (HPRA) | Year of publication: N/A  last update: N/A | Ireland | -understand types of GMP inspections.  -role of HPRA in inspections and how it can be implemented. | -the result of the inspection whether accepted or further deficiencies addressed.  -the issue of GMP certificate.  -provide a map to companies expecting inspections and timeframe to address issues. | Regulatory authority | |
| 4 | <https://ispe.org/initiatives/regulatory-resources/gmp> | Exclude | Exclude | **exclude** | ISPE | Reason provided: It is not mainly relevant to GMP inspections. | | | | |  |
| 5 | <https://safetyculture.com/topics/gmp/> | Include | Exclude | **Exclude** | Safety culture | Reason provided: Not much new information here. | | | | |  |
| 6 | <https://health.ec.europa.eu/medicinal-products/eudralex/eudralex-volume-4_en> | Include | Include | **Include** | EudraLex | Publication: 2011 update:N/A | EU | -what is required for a medicinal product  -guidelines to explain the inspection process and address different aspects of the process | -applying quality risk management approach towards GMP inspections | Policy document | |
| 7 | <https://www.qualio.com/blog/gmp> | Include | Include | **Include** | Qualio | Publication:2024  update:N/A | USA  ireland | -understanding the 5 p’s of GMP  -explore the quality management system  -main things to consider for a GMP compliant company derived from Eudralex | -Compliance benefits  -illustrating different regulatory authorities GMP guidelines | Professional organisation  (private company) | |
| 8 | <https://www.who.int/teams/health-product-policy-and-standards/standards-and-specifications/norms-and-standards/gmp> | Exclude | Include | **include** | WHO | Publication:  update:N/A | USA | -important to know WHO standards as they monitor a lot of eastern countries | -providing GMP guidance according to WHO | International public health agency | |
| 9 | <https://www.fda.gov/drugs/pharmaceutical-quality-resources/current-good-manufacturing-practice-cgmp-regulations> | Exclude | Exclude | **Exlude** | FDA | Reason provided: not enough information on GMP inspections, a broad explanation on GMP, need approval for accessing their provided pdf files regarding GMP. | | | | |  |
| 10 | <https://picscheme.org/docview/6606> | Include | Include | **Include** | PIC/S  Pharmaceutical inspection co-operation scheme | Published 2023  Updated: N/A | switzerland | -harmonise GMP quality standards through provided policy document with chapters elaborating each step of the process | -compliance with manufacturing organisation regarding the marketing of products for their intended use | International organisation | |
| 11 | <https://miaspharma.com/what-are-the-different-types-of-eu-gmp-audits-that-pharma-companies-carry-out/> | Include | Exclude - not much info on this page | **include** | Mias Pharma | Published 2023  Updated: N/A | Ireland | -elaborating on different types of inspection audits  -preparation for an audit as defined by an auditor | -how does audit contribute to GMP inspections compliance whether it is self-inspections or initiated by regulatory authorities. | Professional organisation | |
| 12 | <https://www.onlinegmptraining.com/what-is-a-gmp-inspection/> | Include | Include | **Include** | Pharmaout | Published:2021  update:2022 | Australia | -identify timeframe for GMP compliance audit  -frequently asked questions regarding GMP auditing  -understand requirement for remote auditing. | -addressing audit findings in a timely manner  -hybrid GMP audits (partial onsite and partial desktop auditing method)  -remote audit benefits | Professional organisation | |
| 13 | <https://www.gov.uk/guidance/good-manufacturing-practice-and-good-distribution-practice> | Include | Include | **Include** | Medicine and healthcare products regulatory agency (MHRA) | Published:2014  update:2024 | UK | -how MHRA carry out GMP inspections and perform grading of findings  -elaborate on compliance escalation process | -GMP inspection feedback report was generated to establish findings regarding most common deficiencies found in previous inspections. | Government organisation | |
| 14 | <https://www.gmp-compliance.org/what-is-gmp> | Exclude | Include - | **include** | ECA Academy | Published: N/A  update:N/A | Germany | -provide a quick brief on GMP and its requirements.  -introduce the authorities that is responsible for GMP compliance monitoring in different countries | -the granting of manufacturing authorisation or import permit (non-EU countries)to GMP compliant companies. | Private professional organisation | |
| 15 | <https://ispe.org/topics/gmp> | Exclude | Exclude | **Exclude** | ISPE | Reason provided: not enough information provided on GMP inspections | | | | |  |
| 16 | <https://www.tga.gov.au/how-we-regulate/manufacturing/manufacture-medicine/good-manufacturing-practice-gmp> | Exclude | Exclude | **Exclude** | Australian government department of health and age care | Reason provided: not enough information provided on GMP inspections | | | | |  |
| 17 | <https://www.ease.io/blog/5-main-components-of-good-manufacturing-practice/> | Include | Include | **Include** | Ease Inc. | published:2024  Update: N/A | USA | -introducing the five main components of GMP. | -reduce waste  -prevent mistakes during manufacturing process. | Professional organisation | |
| 18 | <https://slcontrols.com/en/how-good-distribution-practice-gdp-differs-from-good-manufacturing-practice-gmp/> | Exclude | Exclude | **exclude** | SL controls  An nnit group company | Reason provided: provide comparison between two GMP and GDP, unrelated to inspection | | | | |  |
| 19 | <https://www.blog-qhse.com/en/why-good-manufacturing-practices-gmps-are-important-in-the-pharmaceutical-industry> | Include | Exclude | **Exclude** | BlueKanGo | Reason provided: website does not provide additional information | | | | |  |
| 20 | <https://fatfinger.io/gmp-compliance-in-pharmaceutical-manufacturing/> | Include | Include | **Include** | Fat finger | Published:2024  update:N/A | USA | -explain key factors contributing to GMP  - introduce six best practices in pharmaceutical manufacturing | -quality management systems, employee training, and continuous improvement maintain operational efficiency | Professional organisation | |
| 21 | <https://gmpinsiders.com/what-is-good-manufacturing-practices-gmp/> | Include | Exclude - | **Exclude** | GMP insiders | Reason provided: we have all this information already in included sources | | | | |  |
| 22 | <https://key2compliance.com/blog/good-manufacturing-practice-preparing-for-gmp-inspections-and-addressing-common-challenges/> | Include | Include | **Include** | GBA key2compliance | Published:2024  update:N/A | Sweden  Denmark | -how to prepare for an inspection  -addressing GMP challenges | -ensure companies are adhering to protocols and thus improve patient’s safety | Professional organisation | |
| 23 | <https://assets.publishing.service.gov.uk/media/57a08bf9e5274a31e0000eee/60336-WP3.pdf> | exclude | Exclude | **Exclude** | The centre for international public health policy | Reason provided: discuss mainly guidelines and not inspection procedures | | | | |  |
| 24 | <https://www.qualio.com/blog/what-is-cgmp-in-the-pharmaceutical-industry> | exclude | Include - | **include** | Qualio | Publication:2023  update:N/A | USA  Ireland | -expert opinion  -introducing the QMS system for risk-based assessment | - adopting more management systems to enhance compliance | Professional organisation | |
| 25 | <https://iris.who.int/bitstream/handle/10665/64465/WHO_VSQ_97.01-eng.pdf> | Include | Exclude - | **Exclude** | WHO | Reason provided: very long of a source | | | | |  |

| 26 | <https://www.biopharminternational.com/view/beyond-gmps-latest-approaches-good-manufacturing-practices> | Include | Exclude - | **exclude** | BioPharm  International | Reason provided: title of “latest approaches” however it is written in 2004, very outdated source | | | |  |
| --- | --- | --- | --- | --- | --- | --- | --- | --- | --- | --- |
| 27 | <https://www.ease.io/blog/what-are-gmp-guidelines/> | Include | Exclude - | **exclude** | Ease Inc | Reason provided: repetition | | | |  |
| 28 | <https://www.rssl.com/life-science-training-consultancy/life-science-courses/management-of-gmp-inspections/> | exclude | Exclude | **Exclude** | RSSL | Reason provided: a training course for GMP inspection | | | |  |
| 29 | <https://database.ich.org/sites/default/files/Q7%20Guideline.pdf> | Include | Exclude | **Exclude** | ICH | Reason provided: old-dated document (2000) | | | |  |
| 30 | <https://mhrainspectorate.blog.gov.uk/category/good-manufacturing-practice/> | Exclude | Exclude | **Exclude** | MHRA | Reason provided: not enough information on GMP inspection | | | |  |
| 31 | <https://pmc.ncbi.nlm.nih.gov/articles/PMC4399016/> | Include | Include | **Include** | Pubmed academic article | published:2015 | The study is based in Portugal | -a review that maps regulation, production, distribution and consumption of pharmaceutical | -Effective application of the quality control and quality risk management | Academic organisation |
| 32 | <https://www.nsf.org/knowledge-library/the-importance-of-gmp-auditing-services> | exclude | Exclude | **Exclude** | NSF | Reason provided: not enough on GMP inspections | | | |  |
| 33 | <https://www.ideagen.com/thought-leadership/blog/good-manufacturing-practice-compliance-for-life-sciences> | Include | Exclude - | **Exclude** | Ideagen | Reason provided: repetition of what is included above | | | |  |
| 34 | <https://medicinesauthority.gov.mt/goodmanufacturingpractice> | Include | Maybe | **Include** | Malta medicines authority | Publication:N/A  update2024N/A | Malta | -explain types of GMP inspections  -Maltese GMP legislation | - ensuring conditions of manufacturing licence are being met and followed | Government organisation |
| 35 | <https://www.npra.gov.my/easyarticles/images/users/1050/FAQs-APB-Inspection-Feb-2020.pdf> | include | Exclude | **Include but but removed (duplication)** | National Pharmaceutical Regulatory Agency | Publication:N/A  Update: N/A | malaysia | -explain the process flow for setting up GMP for a new manufacturing facility | -non-compliance sanctions and GMP certificate issue |  |
| 36 | <https://www.tga.gov.au/resources/publication/publications/pics-guide-gmp-medicinal-products-version-16> | Exclude | Exclude | **Exclude** | Australian government department of health and age care | Reason provided: not enough information provided on inspection. | | | |  |
| 37 | <https://www.ecfr.gov/current/title-21/chapter-I/subchapter-C/part-211> | Include | Include | **include** | Electronic code of federal regulation | Publication:1978  update2024 | USA | -detailed presentation of GMP requirements in manufacturing facilities during processing distribution, and packaging | -drug product meets all applicable standards of purity, identity and quality | Government organisation |
| 38 | <https://www.onlinegmptraining.com/who-conducts-gmp-audits-regulatory-bodies-worldwide-list/> | Include | Include | **include** | PharmaOut | Publication:2022  updateN/A | Australia | -provide a list of regulatory authorities responsible for GMP inspections  - a list of participating countries in PIC/S  -GMP compliance audit importance  -GMP audit frequently asked questions | -improve worldwide standards of GMP production | Professional organisation |
| 39 | <https://www.tandfonline.com/doi/full/10.1080/20523211.2024.2399722> | Include | Exclude - very narrow research | **Exclude** | Taylor and Frances/journal of Pharmaceutical Policy and Practice/ article | Reason provided: very narrow research | | | |  |
| 40 | <https://pmc.ncbi.nlm.nih.gov/articles/PMC3122044/> | Include | Include | **include** | PubMed academic article | Publication:2011 | India | -regulations review and harmonisation of different GMP requirements and prepare GMP guide for documentation  -the ten golden rules of GMP  -illustrate the hierarchical document system | -checklist provided as a conclusion about documentation and record to help assess compliance in the future | academic organisation |
| 41 | <https://www.gmp-compliance.org/guidelines/gmp-guideline/fda-guidance-for-industry-quality-systems-approach-to-pharmaceutical-current-good-manufacturing-practice-regulations> | Exclude | Exclude | **Exclude** | ECA Academy | Reason provided: not enough information | | | |  |
| 42 | <https://picscheme.org/en/publications> | Exclude | Exclude | **Exclude** | PIC/S | Reason provided: not enough information to cover inspection practices | | | |  |
| 43 | <https://pharmacia.pensoft.net/article/120053/> | Include | Include | **Include but removed (duplication from academic literature)** | Pharmacia journal /articlpe | Published: 2024 | Bulgaria | -assess non-compliance identified during GMP inspections | -impose penalties for non-compliance and highlighting gaps in practices | professional organisation |
| 44 | <https://inpharmatis.com/quality-management/> | Exclude | Exclude | **Exclude** | Inpharmatis | Reason provided: provide consultation service | | | |  |
| 45 | <https://english.nmpa.gov.cn/2019-07/25/c_390613.htm> | Include | Maybe | **Include** | National Medical Products Administration | Published:2011  updated:2019 | China | -provisions of GMP requirements .  -establish quality management system  -prevent contamination and error | -continuous improvement  -patient safety  - improved product quality | government organisation |
| 46 | <https://www.canada.ca/en/health-canada/services/drugs-health-products/compliance-enforcement/good-manufacturing-practices/guidance-documents/gmp-guidelines-0001/document.html> | Include | Exclude | **Exclude** | Health Canada | Reason was not provided | | | |  |
| 47 | <https://amm.atusligo.ie/en/module/web/20/GMP08003/201900/999999> | Exclude | Exclude | **Exclude** | ATU Sligo | Reason provided: compliance auditing academic module | | | |  |
| 48 | <https://www.valicare.com/en/gmp-services/gxp-audits-mock-inspections/> | Exclude | exclude | **Exclude** | Valicare | Reason provided: does not involve GMP inspection practices | | | |  |
| 49 | <https://www.getreskilled.com/pharmaceutical-companies/regulations/> | Exclude | Exclude | **exclude** | Get-Re-Skilled | Reason provided: discuss regulatory authorities more than GMP inspections | | | |  |
| 50 | <https://libsearch.mtu.ie/cgi-bin/koha/opac-detail.pl?biblionumber=48268> | Exclude | Exclude | **exclude** | Munster technological university | Reason provided: a book that needs to be purchased | | | |  |

| 51 | <https://riverark.com/gmp-and-gdp/> | Exclude | Exclude | **exclude** | River ark quality | Reason provided: link not valid | | | |  |
| --- | --- | --- | --- | --- | --- | --- | --- | --- | --- | --- |
| 52 | <https://www.thefdagroup.com/blog/2015/07/gxp-audits/> | Include | Include | **include** | The FDA Group | Published:2022  update:N/A | USA | -manufacturing, packaging, and processing GMP audits | -ensure product is consistent and is of high quality standards | Professional organisation |
| 53 | <https://adamasconsulting.com/good-manufacturing-practice/> | Exclude | Exclude | **exclude** | AADAMAS | Reason provided: audit consulting service | | | |  |
| 54 | <https://ustr.gov/sites/default/files/IssueAreas/Manufacturing/20170119%20Pharma%20MRA%20US%20EU%20%28FINAL%29.pdf> | Exclude | exclude | **exclude** | US-EU amended sectoral annexe | Reason provided: does not contain relevant information regarding GMP inspections | | | |  |
| 55 | <https://www.sciencepharma.com/blog/a-history-of-the-gmp/> | Exclude | exclude | **Exclude** | Science pharma | Reason provided: provide a history of GMP and plant inspections | | | |  |
| 56 | <https://www.rsc.org/events/detail/78648/management-of-gmp-inspections> | Exclude | exclude | **Exclude** | Royal Society of Chemistry | Reason provided: a scheduled event of GMP inspections | | | |  |
| 57 | <https://www.blue-inspection.com/en/gmp-gxp-audits-en> | Include | Exclude | **exclude** | Blue Inspection Body GmbH | Reason was not provided | | | |  |
| 58 | <https://gxp-academy.org/upload/iblock/f41/f4133fb1b470ec68db330bfd5b022be4.pdf> | Include | Exclude | **exclude** | WHO | Reason was not provided | | | |  |
| 59 | <https://resource-allocation.biomedcentral.com/articles/10.1186/s12962-022-00348-1> | Include | Exclude | **Exclude** | Biomedical Central Journal/ article | Reason provided: small sample, we have academic literature paper with much broader one | | | |  |
| 60 | <https://www.susupport.com/knowledge/manufacturing-processes/biopharma/cgmp-everything-need-know> | Include | Exclude - | **exclude** | Single-use support Pioneering  Biopharma | Reason provided: we have all this information already | | | |  |
| 61 | <https://www.sqt-training.com/programme/internal-quality-auditing-for-manufacturers-of-finished-pharmaceuticals/?print=true> | Exclude | exclude | **exclude** | SQT training | Reason provided: a training program | | | |  |
| 62 | <https://fdaghana.gov.gh/wp-content/uploads/2024/10/GUIDELINE-ON-THE-GMP-REQUIREMENTS-FOR-DRUG-MANUFACTURING-FACILITIES.pdf> | Include | Include | **Include** | Food and Drug Authority | Published:2024  Updated: N/A | USA | -guidelines to current GMP practices for drug manufacturing facilities  - emphasise the establishment of quality management system  -increase FDA transparency in inspections . | -consistent production of product that meet the quality standards | government organisation |
| 63 | <https://www.rssl.com/life-science-training-consultancy/life-science-courses/good-manufacturing-practice-the-essentials/> | Exclude | exclude | **exclude** | RSSL | Reason provided: an online course | | | |  |
| 64 | <https://ecvalidation.com/en/how-to-prepare-for-a-gmp-audit/> | Include | Exclude - | **Exclude** | eCValidation | Reason provided: about e-consulting company | | | |  |
| 65 | <https://ascentage.com/ascentage-pharma-passes-gmp-audit-by-eu-qp-with-zero-deficiency/> | exclude | exclude | **Exclude** | Ascentage Pharma | Reason provided: not relevant to GMP inspections | | | |  |
| 66 | <https://www.linkedin.com/pulse/how-prepare-gmp-inspection-5-steps-mehrnaz-bozorgian-nptmf> | Include | Exclude | **Include but removed (duplication)** | Linkedin  / Zamann pharma | Published: 2024  updated: N/A | Germany | -how to prepare for GMP inspections  -providing advice on successful GMP inspections | Successful Navigation of GMP inspection | professional organisation |
| 67 | <https://medicaldeviceregistration.com/blog/what-is-gmp-audit-in-pharma/> | Include | Included | **include** | ELT Corporate Private Limited | Published: N/A  updated: N/A | India | -understand how to prepare for GMP audits,  -non-compliance report outcome  -purpose of GMP inspection  -hybrid inspection method | -performing CAPA to address non-compliance  -increased efficiency due to the introduction of digital tools in auditing process | professional private organisation |
| 68 | <https://www.scilife.io/blog/gmp-standards> | Include | Included | **include** | Scilife | Published: N/A  updated: 2024 | Belgium | -how technology is affecting GMP standards and how is AI contributing to manufacturing  -virtual and augmented reality incorporation into GMP.  -better data sharing through electronic quality management systems | -predict issues before they arise to allow manufacturer to fix it before it damages the product  -revolutionise drug discovery  -better recall management  -less human error  -uninterrupted production | professional organisation |
| 69 | <https://www.direnzo.biz/it/en/pharmaceutical-advice/regulatory-assistance-medicinal-human-use/audit-consulting/> | Exclude | Exclude | **exclude** | Di Renzo regulatory affairs | Reason provided: consulting service | | | |  |
| 70 | <https://www.inspiredpharma.com/wp-content/uploads/2012/02/fda-quality-systems-gmp-guidance.pdf> | Include | Excluded | **Exclude** | Inspired pharma /FDA | Reason provided: Outdated - should have more recent one | | | |  |
| 71 | <https://nafdac.gov.ng/wp-content/uploads/Files/Resources/Guidelines/DRUG_GUIDELINES/NAFDAC-GMP-GUIDELINES.pdf> | Include | Included | **Include** | National agency for food and drug administration and contro  (NAFDAC)l | Published: 2024  updated: N/A | Nigeria | -introducing pharmaceutical quality system principles  -quality control and quality risk management  -each chapter highlights different requirements in GMP inspection process | -providing a guideline to enable manufacturer to comply with the provisions of NAFDAC GMP. | Government organisation |
| 72 | <https://www.nda.or.ug/nda/files/downloads/INS%20GDL%20001-Part%201-GMP%20Guidelines%20for%20Medicinal%20Products_R2.pdf> | Include | Exclude - | **Exclude** | Uganda National Drug Authority | Reason provided: #128 has the same but updated in 2024 | | | |  |
| 73 | <https://www.eurofins.com/assurance/resources/articles/top-five-non-conformities-gmp-audit-pharmaceutical/> | Include | Include | **Include** | Eurofins scientific SE | Published: N/A  updated: N/A | Luxembourg | -identify common non-conformities within the GMP audit systems | -improve performance of pharmaceutical of pharmaceutical companies | professional organisation |
| 74 | <https://www.aifa.gov.it/sites/default/files/2016-05-13_BACCARELLI_13_05_2016_GMP.pdf> | Include | Include | **Include** | AIFA (Italian medicine agency) | Published: 2016  updated: N/A | Italy | -walk through EudraLex legislation and EU GMP legal bases  -Italian manufacturing sites yearly inspections rate  -explain the frequency of inspection | -the assessment of quality system periodically that contribute to GMP compliance | government organisation |
| 75 | <https://www.seerpharma.com/services/qa-and-gmp-consulting/gxp-auditing/gmp> | Exclude | Exclude | **Exclude** | Seer Pharma | Reason provided: introduction about the company | | | |  |

| 76 | <https://www.pharmaceutical-networking.com/pharmaceutical-auditor-training-from-iqc/> | Exclude | Exclude | **Exclude** | Irish Quality centre | Reason provided: audit training services | | | |  |
| --- | --- | --- | --- | --- | --- | --- | --- | --- | --- | --- |
| 77 | <https://www.routledge.com/Good-Pharmaceutical-Manufacturing-Practice-Rationale-and-Compliance/Sharp/p/book/9780367393779?srsltid=AfmBOoq7Uq4Bcg5Kh3rL9OVGMimbjYkfFwVzyu5taoJyNFiHETX8CyP8> | Exclude | Exclude | **Exclude** | Taylor and Francis group | Reason provided: a book that needs to be purchased | | | |  |
| 78 | <https://www.hsa.gov.sg/chinese-proprietary-medicines/dealers-licence/gmp-gdp-standards> | Exclude | Exclude | **exclude** | Health Science Authority | Reason provided: | | | |  |
| 79 | <https://www.anao.gov.au/work/performance-audit/administering-the-code-good-manufacturing-practice-prescription-medicines> | Exclude | Exclude | **Exclude** | Australian National Audit Office  (ANAO) | Reason provided: not highly relevant to GMP inspections | | | |  |
| 80 | <https://www.sciencedirect.com/org/science/article/pii/S0428029624000799> | Include | Include | **Include but removed due to duplication from academic literature** | Science direct/ Pharmacia Journal/article | published:2024 | Bulgaria | -assessing non-compliance in manufacturers during GMP inspections | -penalties due to non-compliance identified in several manufactures | Academic organisation |
| 81 | <https://fessgroup.co.uk/insight/what-is-gmp-inspection/> | Include | Exclude | **Exclude** | Fessgroup | Reason provided: too many overlapping information available | | | |  |
| 82 | <https://www.ifpma.org/areas-of-work/strengthening-regulatory-systems/quality-safety-and-efficacy-of-therapeutical-products/> | Exclude | Exclude | **Exclude** | IFPMA | Reason provided: none was given | | | |  |
| 83 | <https://www.bizzmine.com/en/blog/achieving-gmp-compliance-in-pharma-a-regulatory-guide> | Include | Include | **Include** | Bizzmine | Published: 2025  updated: N/A | Belgium | -introducing the five main components of GMP  -key principles of GMP | -repercussions to non-conformities with GMP compliance  -benefit of eQMS in compliance operations | professional organisation |
| 84 | <https://www.gmp-publishing.com/content/en/gmp-info/what-is-gmp> | Exclude | Exclude | **Exclude** | GMP Verlag | Reason provided: not enough information on GMP inspections | | | |  |
| 85 | <https://www.griffith.ie/sites/default/files/storage/uploads/Faculties/GraduateBusinessSchool/Docs/MSCIPM/Quality%20%26%20Regulatory%20Framework%20in%20the%20Pharmaceutical%20Industry.pdf> | Exclude | Exclude | **Exclude** | Griffith College | Reason provided: module teaching plan | | | |  |
| 86 | <https://iclg.com/briefing/19633-new-agreement-on-the-mutual-recognition-of-good-manufacturing-practice-for-medicinal-products-between-switzerland-and-the-usa> | Exclude | Exclude | **Exclude** | International comparative legal guides | Reason provided: an agreement between Switzerland and USA and is not highly relevant to GMP inspections | | | |  |
| 87 | <https://sahivsoc.org/Files/4.01_sa%20guide%20to%20good%20manufacturing%20practice_jul19_v7.pdf> | Include | Include | **Include** | South African Health Product Regulatory Authority  (SAHPRA) | Published: 2019  updated: N/A | South Africa | -SAHPRA aim in developing international standards between countries and pharmaceutical inspections | -provide a guide to navigating GMP and a list of regulatory authorities | Government organisation |
| 88 | <https://flowdit.com/good-manufacturing-practices/> | Include | Include | **Include** | Flowdit | Published: 2024  updated: N/A | Germany | -importance of GMP, it’s principles  -GMP requirements for pharmaceutical manufacturers | -implementation of robust quality management systems  -high standards of quality and compliance | professional organisation |
| 89 | <https://zamann-pharma.com/2024/04/24/how-to-prepare-for-gmp-inspection-in-5-steps/> | Include | Include | **Include** | Zamann pharma | Published: 2024  updated: N/A | Germany | -how to prepare for GMP inspections  -providing advice on successful GMP inspections | -successful navigation of GMP inspections by pharmaceutical companies | professional organisation |
| 90 | <https://www.micronhvac.com/gmp-audit-in-ireland.html> | Include | Exclude | **Exclude** | Micron HVAC PVT. Ltd. | Reason provided: too many overlapping information available | | | |  |
| 91 | <https://www.gmp-navigator.com/mygmp/validierung-produktion-technik/guidelines-basic-gmp-regulations> | Include | Include | **include** | Concept Heidelberg GmbH | Published: 2013  updated: N/A | Germany | -consolidate various GMP guidelines from authority’s bodies  - provide up-to-date GMP compliance support | -enhance understanding and implementation of GMP standards.  -production of safe and effective pharmaceutical product | professional organisation |
| 92 | <https://www.fda.gov.tw/eng/siteListContent.aspx?sid=10387&id=28055> | Exclude | Exclude | **Exclude** | Taiwan food and drug administration | Reason provided: this website talks more about history of GMP implementation | | | |  |
| 93 | <https://www.chromatographyonline.com/view/current-good-manufacturing-practice-cgmp-an-overview-for-the-analytical-chemist> | Include | Include | **Include** | Chromatography online  LCGC | Published: 2023  updated: N/A | USA | -provide an overview of cGMP regulations, principles and compliance.  -introduce the role of quality control analyst  - provide a blue print for GMP compliant facility  -mutual recognition agreement MRA importance | - quality control analyst are essential to compliance  -adopting quality by design approach during drug development  -‘Consent decree’ court order to address issues and take corrective steps | professional organisation |
| 94 | <https://www.frontiersin.org/journals/public-health/articles/10.3389/fpubh.2023.1103555/pdf> | Include | Include | **Include but removed (duplication from academic literature)** | Frontier journal article | Published: 2023  updated: N/A | China | -provide an insight into inspection and production improvement in China and other GMP compliant companies | empirical study in GMP drug production supervision | Academic organisation |
| 95 | <https://www.regulations.gov/document/FDA-2024-N-4016-0001> | Exclude | Exclude | **Exclude** | FDA | Reason provided: is not highly relevant to GMP inspections | | | |  |
| 96 | <https://gmpinsiders.com/gmp-regulatory-bodies-understand-key-differences/> | Exclude | Exclude | **Exclude** | GMP insider | Reason provided: only talks about mutual recognition agreement and not expand on inspection aspects | | | |  |
| 97 | <https://www.researchgate.net/publication/340324663_Good_Manufacturing_Practice_for_Medicinal_Products_in_Bulgaria_an_Analysis_of_Regulatory_Inspection_Findings> | Include | Include | **Include** | Research gate/ Folia Medica journal/ article | Published: 2020  updated: N/A | Bulgaria | -analyse deficiencies reported during GMP in Bulgaria and compare them to other EU member states | - show that Bulgarian Drug Agency is able to detect non-compliance issues compared to other EU member states  -main reason of non-compliance was found to be quality related.(a trend that is seen in other EU countries) | academic organisation |
| 98 | <https://www.pharmtech.com/view/gmp-gdp-inspections-challenges-and-opportunities-revealed-by-the-covid-19-pandemic> | Include | Include | **Include** | Pharmaceutical  Technology | Published: 2021  updated: N/A | Belgium | -identify how did the COVID-19 pandemic affected inspection  -what did it teach us regarding the need to revolutionise inspections | -virtual inspection issues such as technological and privacy issues, and difficulties with time zone management  -longer time is needed to prepare for virtual inspections | professional organisation |
| 99 | <https://learning.sgs.com/lmt/clmsCatalogDetails.prMain?site=sgsssc&in_region=hk&in_offeringId=44652584&in_language_identifier=en&in_filter=%26in_courseName%3DGMP%26in_location%3D%2525%26in_rows%3D50%26in_courseType%3D%2525%26in_orderBy%3DDA%26in_region%3Dhk%26in_language_logged_out%3Den%26in_start%3D> | Exclude | Exclude | **Exclude** | SGS | Reason provided GMP training course | | | |  |
| 100 | <https://rwandafda.gov.rw/wp-content/uploads/2023/04/Guidelines-on-Good-Manufacturing-Practice-for-Finished-Pharmaceutical-Products-Part-1.pdf> | Include | Include | **Include** | Rwanda Food and Drug Authority | Published: 2023  updated: N/A | Rwanda | -provide internationally accepted guidelines adopted from PIC/S | -major requirements for registration of pharmaceutical products in Rwanda. | Government organisation  (regulatory authority) |

| 101 | <https://www.fda.gov.ph/draft-for-comments-revised-guidelines-on-good-manufacturing-practice-gmp-clearance-for-foreign-drug-manufacturers/> | Exclude | Exclude | **Exclude** | FDA (Philippines) | Reason provided: reason not provided | | | |  |
| --- | --- | --- | --- | --- | --- | --- | --- | --- | --- | --- |
| 102 | <https://onlinelibrary.wiley.com/doi/full/10.1002/puh2.158> | Exclude | Exclude | **Exclude** | Wiley Online Library | Reason was not provided | | | |  |
| 103 | <https://www.eac.int/documents/category/good-manufacturing-practices> | Exclude | Exclude | **Exclude** | EAC | Reason provided: website only provides forms to fill | | | |  |
| 104 | <https://biomedpharmajournal.org/vol11no1/importance-of-self-inspection-in-pharmaceutical-industry-as-per-various-regulatory-guidelines/> | Include | Exclude (Full text not available) | **Include** | Biomedical and Pharmacological Journal | Published: 2018 | India | -requirements of self-inspections by different regulatory authorities  -provide a map for self-inspections | -preventing financial burden on the company  -keeping the good name of the company  -study and consistent quality management systems | Academic  Organisation |
| 105 | <https://arrow.tudublin.ie/cgi/viewcontent.cgi?article=1149&context=level3> | Include | Include | **Include** | TUD | Published: 2020 | Ireland | -introducing pharmaceutical quality system operation  -provide a roadmap to self-inspection process | -management commitment to self-inspections  -checklists of change management, CAPA, self-inspections, management review. | Academic  Organisation |
| 106 | <https://english.cbg-meb.nl/topics/bd-manufacturing-f-gmp> | Exclude | Exclude ( | **Exclude** | Reason provided: It’s about veterinary medicine | | | | |  |
| 107 | <https://www.complianceonline.com/resources/3-common-violations-of-cgmp-regulations-for-finished-pharmaceuticals-and-how-to-prevent-them.html> | Include | Include | **Include** | Compliance Online | No specific publication date | USA | -provide an overview of three most common cGMP violations | -examples of warning letters issued by the FDA due to non-compliance | Professional organisation |
| 108 | <https://pharmuni.com/2024/07/01/exploring-the-impact-of-gmp-compliance-on-the-pharmaceutical-industry-a-comprehensive-guide-for-specialists/> | Include | Include | **Include** | Pharmuni | Published in: 2024  Updated:  N/A | Germany | -Understanding key concepts in GMP  -role of technology in GMP inspections | - rigorous adherence to GMP protect companies from regulatory and financial loses  -globalisation of GMP standards  - | Professional organisation |
| 109 | <https://www.pharmaceutical-technology.com/features/pharma-inspections/> | Include | Include | **Include** | Pharmaceutical Technology | Published :2020  Updated: N/A | UK | -introducing inspectors’ role  -understanding when inspections occur | -inspection report is generated (general information, description of inspection, observation and conclusion) | Professional organisation |
| 110 | <https://www.apprentice.io/life-science-glossary/gmp-good-manufacturing-practice> | Exclude | Exclude | **Exclude** | Reason provided: sufficient information on this topic already- what is GMP etc. | | | | |  |
| 111 | <https://www.federalregister.gov/documents/2024/09/20/2024-21559/revocation-of-regulations-regarding-the-mutual-recognition-of-pharmaceutical-good-manufacturing> | Exclude | Exclude | **Exclude** | Reason provided: legal regulations that is not relevant | | | | |  |
| 112 | <https://www.ich.org/page/quality-guidelines> | Include | Included | **Include** | International council for harmonisation (ICH) | Published:  Updates | Switzerland | -overview of GMP requirements such as sterility, stability, and analytical validation | -improvement in GMP risk management | Non-profit organisation |
| 113 | <https://www.caq.de/en/solutions/gmp-software> | Include | Included | **Include** | CAQ AG factory systems | 2015 | Germany | -Provide the insights that we don’t have about GMP software for inspection | -different software that can be used in different parts of the inspection process such as CAPA and root cause analysis |  |
| 114 | <https://www.mastercontrol.com/au/manufacturing/gmp/compliance/> | Exclude | Excluded | **Exclude** | Masters control | Reason provided: a book to be purchased | | | |  |
| 115 | <https://boracdmo.com/what-are-gxps-and-how-do-they-shape-the-quality-of-your-pharmaceutical-product/> | Exclude | Excluded | **Exclude** | Bora pharmaceuticals | Reason provided: not relevant to GMP inspections | | | |  |
| 116 | <https://www.iqc.ie/training/pharmaceutical-training/> | Exclude | Excluded | **Exclude** | IQC training, consultancy, and auditing | Reason provided: training program | | | |  |
| 117 | <https://www.propharmagroup.com/thought-leadership/navigating-eu-gmp-compliance-guide> | Include | Included | **Include** | Pro-pharma | Published 2024  Updated/A | UK | -specify the real-world problems in GMP inspection | -stay updated  -regular audits  - foster a quality culture | Professional organisation |
| 118 | <https://www.tmda.go.tz/pages/gmp-inspection> | Exclude | Excluded | **Exclude** | Tanzania medicine and medical device authority | Reason provided: does not elaborate on GMP inspections heavily | | | |  |
| 119 | <https://ossmideast.com/good-manufacturing-practice-gmp/> | Exclude | Excluded | **Exclude** | OSS middle eastern certification | Reason provided: mostly about cGMP and not inspections | | | |  |
| 120 | <https://www.wjpmr.com/download/article/121042024/1714381499.pdf> | Exclude | Include | **Include** | World journal of pharmaceutical and medical research | Published:2024 | India | -importance of GMP in the healthcare sector  -fundamental tenets of GMP are explored  -addressing difficulties of GMP implementation in the future | -quality control guarantee safety, efficacy and quality of it’s healthcare goods. | Academic organisation |
| 121 | <https://www.pharmout.net/good-documentation-practices-gdocp-gmp/> | Exclude | Excluded | **Exclude** | Pharma Out | Reason was not provided | | | |  |
| 122 | [4768](https://picscheme.org/docview/4768) | Include | Included | **Include** | PIC/S | Published : 2022  Updated: N/A | Switzerland | -provide an initiative to engage with international organisations  -global harmonisation of GMP standard | -promote and improve inspection reliance  -invest in technology to support it | International cooperative organisation |
| 123 | <https://www.compliancequest.com/quality/gmp-quality/> | Exclude | Excluded | **Exclude** | Compliance quest | Reason was not provided | | | |  |
| 124 | <https://www.tuv-nord.com/in/en/blog/blog-details/article/current-good-manufacturing-practices-cgmp-in-pharmaceutical-industry/> | Exclude | Excluded | **Exclude** | TUV India | Reason provided: not sufficient information on GMP | | | |  |
| 125 | <https://www.scilife.io/blog/main-components-gmp> | Included | Included | **Include** | Scilife | Published :2025 | USA | -discuss GMP guidelines and quality management systems  -introduce the five p’s of GMP | -advocation for continuous GMP improvement | Private company |

| 126 | <https://www.thefdagroup.com/blog/gmp-audits> | Include | Included | **Include** | The FDA Group | Published: 2022 updated: N/A | USA | -introducing the concept of ‘war room’  -how do companies prepare for an inspection | -the audit process provides a picture of how well the company is operating | Private consulting firm |
| --- | --- | --- | --- | --- | --- | --- | --- | --- | --- | --- |
| 127 | <https://www.hpra.ie/regulation/veterinary-medicines/manufacture-of-veterinary-medicines-in-ireland/gmp-inspections-(veterinary-medicines)> | Exclude - | Excluded | **Exclude** | HPRA | Reason provided: Veternary use | | | |  |
| 128 | <https://www.nda.or.ug/wp-content/uploads/2024/07/INS-GDL-001_Part-1_GMP-Guidelines-for-Medicinal-Products_R5.pdf> | Included | Included | **Include** | Uganda National Drug Authority | Published: 2024 | Uganda | -2024 version of Uganda GMP inspection guideline - #72 has the same but outdated  - align Uganda GMP standards with other international regulatory authorities | -improve public health  -improve product quality | Regulatory authority |
| 129 | <https://www.pharmcouncil.co.za/Media/Default/Documents/SA%20Guide%20to%20GMP%20June%202010.pdf> | Exclude | Excluded | **Exclude** | Pharma council | Reason provided: Africa GMP inspection guideline- outdated/2010 | | | |  |
| 130 | <https://miaspharma.com/gmp-requirements-in-the-european-union/> | Include | Excluded | **Include** | miaspharma | Published:2019  Updated: N/A | Ireland | -audit preparation from an auditor’s perspective  -principles of GMP requirements in the EU | -keeping patient safety at the forefront. | Professional organisation |
| 131 | <https://www.intertek.com/pharmaceutical/auditing/> | Exclude | Excluded | **Exclude** | Intertek  Total quality assured | Reason provided: not enough information on GMP inspections | | | |  |
| 132 | <https://www.basg.gv.at/en/inspections/gmp/-gdp> | Exclude | Excluded | **Exclude** | Austrian federal office for safety in healthcare | Reason provided: not enough information on GMP inspections | | | |  |
| 133 | <https://www.lexology.com/library/detail.aspx?g=028dd974-5ac6-4c83-b5b6-df4f3af00ed0> | Exclude | Excluded | **Exclude** | Lexology library | Reason provided: not enough information on GMP inspections | | | |  |
| 134 | <https://amconsultancy.be/pharmaceutical-gmp-audits/> | Exclude | Excluded | **Exclude** | Am consultancy | Reason provided: consultancy company | | | |  |
| 135 | <https://www.medsafe.govt.nz/regulatory/guideline/PE_009-8_GMP_Guide%20_Part_I_Basic_Requirements_for_Medicinal_Products.pdf> | Exclude | Include | **Include** | medsafe | Published: 2009  Updated:N/A | New Zeeland | -harmonization of regulatory guidelines  -provide chapters elaborating on key aspects of the inspection process | -enhanced patient safety and satisfaction | Regulatory authority |
| 136 | <https://libsearch.mtu.ie/cgi-bin/koha/opac-detail.pl?biblionumber=116585> | Exclude | Excluded | **Exclude** | MTU library | Reason provided: can’t access the provided book | | | |  |
| 137 | <https://www.canada.ca/en/health-canada/services/drugs-health-products/compliance-enforcement/good-manufacturing-practices.html> | Exclude | Excluded | **Exclude** | Government of Canada. | Reason provided: not related to GMP inspections | | | |  |
| 138 | <https://www.sciencedirect.com/science/article/pii/S2949866X24001060> | Exclude | Excluded | **Exclude** | Science direct | Reason was not provided | | | |  |
| 139 | <https://www.raps.org/products/good-manufacturing-practice-gmp-40-rac> | Exclude | Excluded | **Exclude** | Regulatory affairs professional society | Reason provided: not related to GMP inspections | | | |  |
| 140 | <https://mhrainspectorate.blog.gov.uk/2024/02/20/15076/> | Exclude | Excluded | **Exclude** | Mhra insectorate | Reason provided: relevant but not enough information | | | |  |
| 141 | <https://journals.sagepub.com/doi/10.1177/009286159402800408?icid=int.sj-full-text.similar-articles.9> | Exclude | Excluded | **Exclude** | sagepub | Reason provided: it would have been good if we had the full text access since talking about GMP overview and actual status/ no access | | | |  |
| 142 | <https://www.therqa.com/knowledge-hub/areas-of-interest/good-manufacturing-practice/regulations-guidelines/> | Exclude | Excluded | **Exclude** | The RQA | Reason provided: not related to GMP inspections | | | |  |
| 143 | <https://www.bionity.com/en/encyclopedia/Good_Manufacturing_Practice.html> | Exclude | Excluded | **Exclude** | Bionity | Reason provided: not related to GMP inspections | | | |  |
| 144 | <https://www.tandfonline.com/doi/pdf/10.1080/20523211.2024.2399722> | Exclude | Include | **Include** | Taylor and Frances/ journal of pharmaceutical policy and practice | Published:2024 | UK/ study was conducted in Tanzania | -Good focus on inspection processes and impact of external factors like pandemics  - compare GMP conformance of manufacturers across two years | -importance of corrective actions  - importance of inspections for high standards quality products | Professional organisation |
| 145 | <https://simplerqms.com/quality-management-system-gmp/> | Include | Included | **Include** | Simpler QMS | Published: 2023  Updated: 2025 | USA  Philippines  Denmark | -defining GMP and its importance  - introducing different GxP terms | -improving customer satisfaction  -protect product from adulteration | Private Company |
| 146 | <https://learn.marsdd.com/article/good-manufacturing-practices-gmp-drugs-and-biologics/> | Exclude | Excluded | **Exclude** | marsdd | Reason was not provided | | | |  |
| 147 | <https://zamann-pharma.com/glossary/audit-and-inspection/> | Include | Exclude (info covered in other sources) | **Include** | Zamann | Published:N/A | Germany | -introducing applications of audits  -principles or methods of audits | -ensure compliance with quality standards | Professional organisation |
| 148 | <https://www.micronhvac.com/gmp-training-in-ireland.html> | Exclude | Excluded | **Exclude** | microhvac | Reason provided: training course | | | |  |
| 149 | <https://www.linkedin.com/posts/dr-dhriti-gxp-expert_the-impact-of-regulatory-inspections-on-pharmaceutical-activity-7262290610462576640-lnMG> | Exclude | Excluded | **Exclude** | Linkedin | Reason was not provided | | | |  |
| 150 | <https://www.thermofisher.com/ie/en/home/biotech-lab-solutions/biotech-learning-center/ruo-gmp-compliant-biologics.html> | Exclude | Excluded | **Exclude** | ThermoFisher | Reason was not provided | | | |  |

| 151 | <https://en.wikipedia.org/wiki/Good_manufacturing_practice> | Exclude | Excluded | **Exclude** | Wikipedia | Reason was not provided | | | |  |  |
| --- | --- | --- | --- | --- | --- | --- | --- | --- | --- | --- | --- |
| 152 | <https://www.npra.gov.my/easyarticles/images/users/1133/2023%20Apr_updateDec23/Frequently-Asked-Questions-GMP-Inspection---April-2023.pdf> | Exclude | Include | **Include** | National pharmaceutical regulator authority | Published  Updated | Malaysia | -explain the process flow for setting up GMP for a new manufacturing facility  -addressing frequently asked questions | -encourage compliance and address queries | | Regulator authority |
| 153 | <https://www.ecfr.gov/current/title-21/chapter-I/subchapter-A/part-26> | Exclude | Excluded | **Exclude** | Code of federal regulations | Reason was not provided | | | | |  |
| 154 | <https://www.cfpie.com/how-gmp-best-practices-ensure-quality-control-in-the-pharmaceutical-industry> | Include | Included | **Include** | CfPIE | Published: 2023  Updated:N/A | USA | -provide an overview of guidelines and regulations  -importance of GMP practice and training | -consistent product quality  - better economic and operational efficiency  - better patient trust | | Professional organisation |
| 155 | <https://www.ideagen.com/thought-leadership/blog/gxp-compliance-in-the-pharmaceutical-industry> | Exclude | Excluded | **Exclude** | Ideagen | Reason was not provided | | | | |  |
| 156 | <https://www.valicare.com/en/gmp-services/> | Exclude | Excluded | **Exclude** | Valicare | Reason provided: not that relevant to GMP inspections | | | | |  |
| 157 | <https://globalizationandhealth.biomedcentral.com/articles/10.1186/s12992-015-0110-3> | Exclude | Excluded | **Exclude** | Globalization and health | Reason was not provided | | | | |  |
| 158 | <https://www.nafdac.gov.ng/wp-content/uploads/Files/Resources/Guidelines/DRUG_GUIDELINES/NAFDAC-GMP-GUIDELINES-FOR-PHARMACEUTICAL-PRODUCTS-2016.pdf> | Exclude | Excluded | **Exclude** | NAFDAC | Reason was not provided | | | | |  |
| 159 | <https://www.researchgate.net/publication/384191566_Good_manufacturing_practice_inspections_conducted_by_Tanzania_medicines_and_medical_devices_authority_a_comparative_study_of_two_fiscal_years_from_2018_to_2020> | Exclude | Excluded | **Include**  **But removed due to duplication** | Research gate/academic journal | Removed due to duplication | | | | |  |
| 160 | <https://www.complianceonline.com/resources/good-manufacturing-practices.html> | Exclude | Excluded | **Exclude** | Compliance online | Reason was not provided | | | | |  |
| 161 | <https://www.eurofins.com/assurance/industry/pharmaceutical/> | Exclude - | Excluded | **Exclude** | Eurofins | Reason provided: limited information more aimed at companies | | | | |  |
| 162 | <https://www.hsa.gov.sg/therapeutic-products/register/gmp-conformity-assessment> | Exclude | Excluded | **Exclude** | HSA | Reason provided: not sufficient information regarding inspection | | | | |  |
| 163 | <https://www.gmp-navigator.com/files/guidemgr/WC500004706.pdf> | Exclude - | Excluded | **Exclude** | EMA | Reason provided: more on procedures on inspections than on how to manage them | | | | |  |
| 164 | <https://www.pharmout.net/pic-s-gmps-vs-who-gmps-whats-the-difference/> | Include | Included | **Include** | Pharma-Out | Published:N/A  Updated:2023 | Australlia | -comparison between WHO and PIC/S GMP  -identifying discrepancies between the two | -overlap in certain sections between the two | | Professional organisation |
| 165 | <https://www.fda.gov.tw/eng/siteListContent.aspx?sid=10387&id=28056> | Exclude - | exclude | **Exclude** | FDA | Reason provided: not highly relevant | | | | |  |
| 166 | <https://www.propharmagroup.com/services/quality-and-compliance/gmp-audit-gmp-consulting/> | Exclude - | exclude | **Exclude** | Propharma group | Reason provided: consulting company website | | | | |  |
| 167 | <https://www.mastercontrol.com/gxp-lifeline/fda-quality-system-guidance-pharma-industry/> | Exclude | Exclude | **Exclude** | Masters control | Reason was not provided | | | | |  |
| 168 | <https://www.griffith.ie/sites/default/files/storage/uploads/Module%2011%20-%20Quality%20and%20Regulatory%20Practice%20in%20the%20Pharma%20Industry.pdf> | Exclude | exclude | **Exclude** | Griffith | Reason provided: a training module | | | | |  |
| 169 | <https://www.yokogawa.com/news/press-releases/2025/2025-02-03/> | Exclude | exclude | **Exclude** | yokogawa | Reason provided : not highly relevant | | | | |  |
| 170 | <https://www.bwhealthcareworld.com/article/gland-pharma-gets-2-observations-on-form-483-in-a-pre-market-inspection-by-usfda-489088> | Exclude | exclude | **exclude** | BW Healthcare work | Reason provided : not highly relevant to inspections | | | | |  |
| 171 | <https://www.lexology.com/library/detail.aspx?g=66a637b1-e05b-4a8b-b5f4-79d321c5074d> | Exclude | exclude | **Exclude** | lexology | Reason provided: does not contain any information on GMP | | | | |  |
| 172 | <https://www.fda.gov.ph/downloadables/> | Exclude | exclude | **exclude** | FDA | Reason provided: does not contain any information on GMP | | | | |  |
| 173 | <https://www.bwhealthcareworld.com/article/sun-pharma-gets-usfda-warning-letter-for-halol-facility-regulator-warns-action-461274> | Exclude | exclude | **Exclude** | BWhealthcare world | Reason provided: does not contain any information on GMP | | | | |  |
| 174 | <https://www.pci.nic.in/PolicyCircular.html> | Exclude | exclude | **exclude** | Pci.nic | Reason provided: does not contain any information on GMP | | | | |  |
| 175 | <https://www.akingump.com/en/insights/articles/2025-perspectives-in-private-equity-health-care-and-life-sciences> | exclude | exclude | **exclude** | akingump | Reason provided: does not contain any information on GMP and talks about other healthcare aspects | | | | |  |

| 176 | <https://www.indiainfoline.com/news/business/alembic-pharmas-vadodara-plant-gets-vai-status-from-us-fda> | Exclude | exclude | **exclude** | India Infoline | Reason provided: does not contain any information on GMP | | | |  |
| --- | --- | --- | --- | --- | --- | --- | --- | --- | --- | --- |
| 177 | <https://www.pharmaceuticalonline.com/solution/logistics> | Exclude | exclude | **Exclude** | Pharmaceutical online | Reason provided: does not contain any information on GMP | | | |  |
| 178 | <https://coursevania.com/courses/pharmaceutical-quality-control/> | Exclude | exclude | **exclude** | course Vania | Reason provided: a training course | | | |  |
| 179 | <https://www.pci.nic.in/> | Exclude | exclude | **exclude** | Pci.nic | Reason provided: does not contain any information on GMP | | | |  |
| 180 | <https://www.pharmaceuticalonline.com/topic/cleanroom> | Exclude | exclude | **exclude** | Pharmaceutical online | Reason provided: does not contain any information on GMP | | | |  |
| 181 | <https://hk.jobsdb.com/lab-tester-jobs/in-Mong-Kok-Yau-Tsim-Mong-District/full-time> | Exclude | exclude | **exclude** | jobs dB | Reason provided: a job advertisement | | | |  |
| 182 | <https://www.gowork.pl/oferta/qc-microbiologist,5e99pJVSuuqplJKBKeExFs,exton> | Exclude | exclude | **exclude** | Go work | Reason provided: a different language that is not English | | | |  |
| 183 | <https://en.wikipedia.org/wiki/Milk_allergy> | Exclude | exclude | **exclude** | wikipedia | Reason provided: does not contain any information on GMP and talks about milk allergy | | | |  |
| 184 | <https://www.thefdagroup.com/blog/gmp-audits> | Include - | exclude | **Include but removed due to duplication** | The FDA group | Removed | | | |  |
| 185 | <https://www.thefdagroup.com/blog/how-to-prepare-for-an-fda-inspection> | Include - | include | **included but removed (duplication)** | the FDA group | Removed | | | |  |
| 186 | <https://zamann-pharma.com/2024/04/24/how-to-prepare-for-gmp-inspection-in-5-steps/> | Include - | include | **include but removed (duplication)** | Zaman | Removed | | | |  |
| 187 | <https://www.scilife.io/blog/gmp-standards> | Include | Include | **include but removed (duplication)** | Scilife | Removed | | | |  |
| 188 | <http://www.it-asso.com/gxp/eudralex_v27/contents/homev4.htm> | Include | include | **include** | Eudralex | Published: 2003  Updated:N/A | EU | -provide a guideline on GMP practices and cover all aspects of inspections in volume four | -provide chapters about the rules governing medicinal products in the European union | Regulatory authority |
| 189 | <https://onlinelibrary.wiley.com/doi/full/10.1002/puh2.158> | Include | include | **include** | Online library /journal article | Published: 2024 | USA/ study happened in Australia and Bhutan | -provide an overview on barriers to GMP compliance such as shortage of shortage of skilled people and lack of training opportunities | -optimise available resources in low- and middle-income countries | Publishing private company |
